# Assessing the effect of pregnancy intention at conception on the continuum of care in maternal healthcare services use in Bangladesh: Evidence from a nationally representative cross-sectional survey

**DOI:** 10.1371/journal.pone.0242729

**Published:** 2020-11-20

**Authors:** Md Nuruzzaman Khan, Melissa L. Harris, Deborah Loxton

**Affiliations:** 1 Department of Population Sciences, Jatiya Kabi Kazi Nazrul Islam University, Trishal, Mymensingh, Bangladesh; 2 Faculty of Health and Medicine, School of Medicine and Public Health, University of Newcastle, Newcastle, Australia; 3 Faculty of Health and Medicine, Priority Research Centre for Generational Health and Ageing, School of Medicine and Public Health, University of Newcastle, Newcastle, Australia; Johns Hopkins Bloomberg School of Public Health, UNITED STATES

## Abstract

**Background:**

The Continuum of Care (CoC; defined as accessing the recommended healthcare services during pregnancy and the early postpartum period) is low in lower-middle-income countries (LMICs). This may be a major contributor to the high rates of pregnancy-related complications and deaths in LMICs, particularly among women who had an unintended pregnancy. With a lack of research on the subject in Bangladesh, we aimed to examine the effect of unintended pregnancy on CoC.

**Methods:**

Data from 4,493 mother-newborn dyads who participated in the cross-sectional 2014 Bangladesh Demographic and Health Survey were analysed. Women’s level of CoC was generated from responses to questions on the use and non-use of three recommended services during the course of pregnancy: four or more antenatal care (ANC) visits, skilled birth attendance (SBA) during delivery, and at least one postnatal care (PNC) visit within 24 hours of giving birth. Global recommendations of service use were used to classify CoC as high (used each of the recommended services), moderate (used at least two of the three recommended services), and low/none (no PNC, no SBA, and ≤3 ANC visits). Women’s pregnancy intention at the time of conception of their last pregnancy (ending with a live birth) was the major exposure variable, classified as wanted, mistimed, and unwanted. Unadjusted and adjusted (with individual-, household-, and community-level factors) multilevel multinomial logistic regression models were used to assess the association between unintended pregnancy and level of CoC.

**Results:**

In Bangladesh, the highest level of CoC occurred in only 12% of pregnancies that ended with live births. This figure was reduced to 5.6% if the pregnancy was unwanted at conception. The antenatal period saw the greatest drop in CoC, with 65.13% of women receiving at least one ANC visit and 26.32% having four or more ANC visits. Following the adjustment of confounders, an unwanted pregnancy was found to be associated with 39% and 62% reduced odds of women receiving moderate and high levels of CoC, respectively, than those with a wanted pregnancy. Having a mistimed pregnancy was found to be associated with a 31% reduction in odds of women achieving a high CoC than women with a wanted pregnancy.

**Conclusion:**

Almost nine in ten women did not achieve CoC in their last pregnancy, which was even higher when the pregnancy was unintended. Given that the ANC period has been identified as a critical time for intervention for these women, it is necessary for policies to scale up current maternal healthcare services that provide in-home maternal healthcare services and to monitor the continuity of ANC, with a particular focus on women who have an unintended pregnancy. Integration of maternal healthcare services with family planning services is also required to ensure CoC.

## Introduction

Reducing complications in pregnancy and childbirth remains a major challenge in low- and middle-income countries (LMICs). Complications are responsible for around 822 maternal deaths a day in LMICs (99% of the 830 maternal deaths worldwide) [1,2]. Moreover, of the 5.4 million under-five deaths, which occur globally every year, 4.4 million deaths could be averted if LMICs had the same under-five mortality rate (currently 69 deaths per 1,000 live births) as high-income countries (5 deaths per 1,000 live births) [3]. To do so, the current under-five mortality rate in LMICs needs a 14-fold reduction [3]. The current neonatal mortality (2.5 million annually in LMICs; 47% of the total under-five deaths) has to be reduced by around 50 times, most of which occur because of birth complications [4,5]. Therefore, maternal, newborn and child health remains a priority issue in LMICs [6–8]. Consequently, Sustainable Development Goal (SDG) 3.1 (reduce maternal mortality ratio to less than 70 per 100,000 live births) and SDG 3.2 (reduce under-five and neonatal deaths to 25 and 12 per 1,000 live births, respectively) were designed to significantly reduce maternal and under-five deaths between 2015 and 2030 [9]. Achieving these ambitious goals requires ensuring universal access to sexual and reproductive healthcare services (SDG 3.7) and universal health coverage (SDG 3.8) for women and children under five, which are still challenges in LMICs [3,9].

Elements of the Millennium Development Goals (MDG) between 2000 and 2015 focused on contraception, antenatal care (ANC), and skilled birth attendance (SBA) coverage [10]. The targets were a two-thirds reduction of under-five mortality (MDG 4) and a three-quarters reduction of maternal mortality (MDG 5) [10]. Consequently, global healthcare services coverage was increased significantly (e.g. SBA increased from 59% in 1990 to 71% in 2015) although not in LMICs, including Bangladesh [2,10,11]. Increased coverage of particular healthcare services does not ensure the prevention of maternal, newborn and child mortality [12]. Consequently, MDGs 4 and 5 were mostly unmet in LMICs [2]. Also important was an inadequate focus on postnatal care (PNC) in the MDGs, which global estimates found could be effective in preventing approximately 80% of current maternal deaths and two-thirds of current neonatal deaths [8,13]. Therefore, the World Health Organization (WHO) is now advocating for integrated service delivery for women and children from pre-pregnancy to delivery and the immediate postnatal period [14,15], whereas the SDGs are prioritizing universal health coverage [9].

Continuum of Care (CoC) focuses on the continuation of care received throughout the life cycle and the integration of health providers at various level of services (primary, secondary, and tertiary levels). CoC throughout pregnancy is focused on the use of at least four skilled ANC visits during pregnancy (later changed to eight ANC visits in 2016), SBA during delivery, and the first PNC visit for women and their newborns within 24 hours of birth. The WHO recommends these services to achieve better pregnancy outcomes and significant reductions in maternal and newborn morbidity and mortality in LMICs [9]. The pathway to such reductions is through preparing women for delivery [16], by enabling them to identify illnesses [17], by enabling healthcare facilities to provide obstetric care [18], and by referring women to a higher level of services to avoid life-threatening obstetric complications [17].

The available evidence has shown there is a low rate of CoC and little has changed in LMICs, especially during the period of the MDGs [12,18–20]. One important reason for this low rate is the lack of focus on family planning and preconception care in CoC services. This is a missed opportunity to start women on the CoC pathway, with many prevention activities needing to occur prior to conception. Women with an unintended pregnancy are at particular risk of not receiving CoC as unintended pregnancy often occurs in the absence of family planning and preconception care [21]. Despite this, it is still unclear where the gaps are in seeking care along the continuum and what factors contribute to these gaps [12,19]. The few studies conducted on this topic are equivocal, particularly in terms of estimated CoC rates and what factors contribute to a lack of CoC [12,18–20].

Two studies from retrospective cross-sectional surveys in Ghana found that around 8% of pregnant women received CoC. Here, CoC was associated with women’s geographical location, cohabiting marital status, traditional religion, transportation problems, and lack of knowledge regarding childhood illnesses [20,22]. Similar factors were found in an analysis of nine countries in sub-Saharan Africa (13.9% CoC) and South Asia (24.5% CoC), where 85.5% of the current global maternal deaths occur [12]. Meanwhile, increased age at first delivery, lower parity, increased years of education and wealth quintile, residing in urban areas, having high autonomy, and exposure to mass media were factors found to be associated with 27% and 45% completion of CoC in Pakistan [18] and Nepal [23], respectively. These factors were also found to be common in a regionally-based study conducted in Tanzania, where 10% of women had CoC [24]. However, in Cambodia, of these factors, only being in the highest wealth quintile was found to be significantly associated with a 60% CoC rate [19]. Importantly, these estimates of CoC are significantly lower than comparable countries’ weighted indicators (composite coverage index) of ANC, SBA and PNC [25]. This further suggests that achieving CoC is associated with a different set of factors than the factors associated with particular service use, as receiving a service at one level does not necessarily mean the following service is received.

The high occurrence of obstetric complications challenges the achievement of the SDGs’ targets of reducing maternal and newborn deaths in LMICs and Bangladesh [26]. Obstetric complications are even higher for women with an unintended pregnancy, who represent around 43% of total pregnancies at conception in Bangladesh (including pregnancies that are terminated) and around one-quarter of the total pregnancies that ended with live births [26–28]. This may be a result of not using components of CoC or discontinuation of CoC. However, previous research identified the negative effects of unintended pregnancy on specific service use (ANC, SBA and PNC) in LMICs, including Bangladesh [21,27,29,30]. The causes are individual correlates of unintended pregnancy (e.g. lower education, lower wealth quintile), family influences (e.g. husband and family member opposition and negligence), community context (e.g. higher poverty and lower education), as well as psychological responses following pregnancy (e.g. depression and anxiety) [21,26]. However, focus on the effect of unintended pregnancy on CoC is notably absent in the global literature. To the best of our knowledge, only two studies in Ghana have examined the effect of unintended pregnancy on CoC. Both were conducted in a regional context, where a positive association [20] and non-association [22] with CoC were found, respectively, for women reporting a mistimed and unintended pregnancy compared to women with a wanted pregnancy. These differing findings are in part due to the adjustment of different confounders and the analysis of small samples. Therefore, the objective of this study was to examine the effect of pregnancies that were unintended at conception on the level of CoC in Bangladesh. Nationally representative samples were analysed, controlling for a range of individual-, household-, and community-level factors that may potentially influence the association.

## Methods

### Study design

This study analysed secondary data extracted from the 2014 Bangladesh Demographic and Health Survey (BDHS). The details of the survey have been published elsewhere [11,31]. In brief, the BDHS was a nationally representative cross-sectional survey conducted in all sampled households, where women aged 15–49 years were eligible to participate in an individual questionnaire. The households were selected based on a stratified two-stage cluster design whereby the first stage was the sample enumeration areas or clusters drawn from the 2011 Bangladesh national census framework. A sample of households was selected in the second phase through stratified random sampling of the list of households in each selected cluster. The National Institute of Population Research and Training in Bangladesh conducted this survey in collaboration with MEASURE DHS, USA. Financial and technical support was provided by international development organizations, including The United States Agency for International Development (USAID) and The United Nations Children’s Fund (UNICEF) [11].

### Data

**Fig 1:**
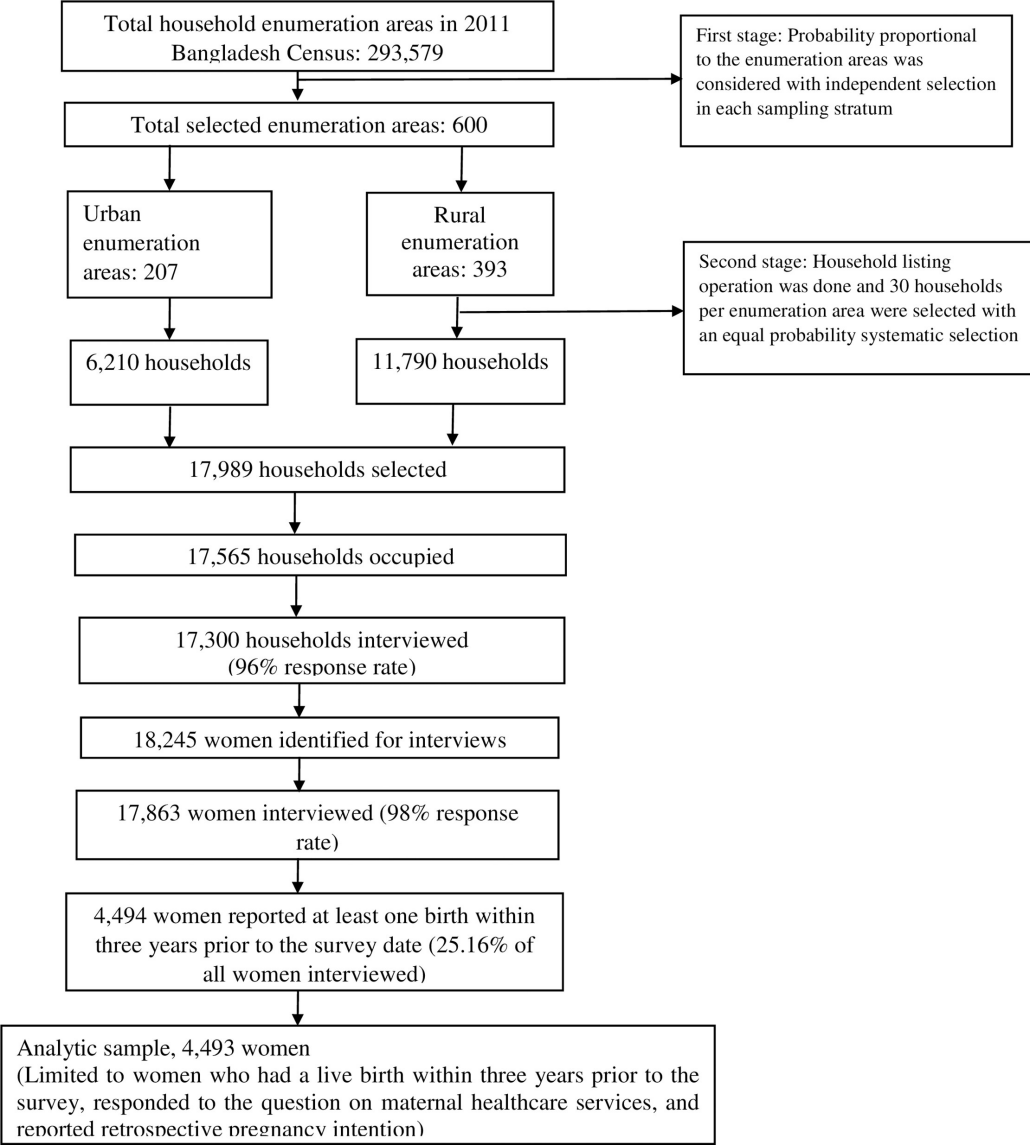
Schematic of analytic sample selection process for unintended pregnancy and continuity of care of maternal healthcare services.

Data from a subsample of 4,493 of the 17,863 women who took part in the survey were analysed. Details of this sample selection procedure are presented in Fig 1. The conditions of inclusion were as follows: i) provided a response to the retrospective question regarding giving live birth in the three years prior to the date of the survey; ii) responded to the questions on the use of maternal healthcare services during their pregnancy (most recent pregnancy if more than one pregnancy occurred); and iii) reported on pregnancy intention at conception of the included pregnancy (which was reported retrospectively*)*.

### Outcome variable

The outcome variable, CoC, was generated from women’s responses to questions on the use or non-use of ANC, SBA, and PNC. Two survey questions regarding ANC services were used to determine if women had received ANC, and if so, how many times. Each eligible woman was first asked, “Did you see anyone for antenatal care for this pregnancy?”. Women who responded “Yes” to this item were then asked, “How many times did you receive antenatal care during this pregnancy?”. SBA status was derived by combining women’s responses to the two questions that were asked to determine who had provided care during delivery (“Who assisted with the delivery of [name of the most recent child born in the past three years])?” and where delivery had occurred (“Where did you give birth to [name of the most recent child born in the past three years])?”. Four additional questions collected information on the timing of the first PNC visit for women and newborns, as well as providers of PNC. Each eligible woman was first asked, “Did anyone check on you and your baby’s health following birth?” Further questions were asked to determine the timing (“How long after delivery did the first check take place?”), place (“Where did this first check take place?”), and providers (“Who checked on your health at that time?”) of PNC. These items were recoded to generate the CoC variable depending upon the continuity of using maternal healthcare through to PNC. The WHO guidelines in 2014 (which were in use at the time of survey completion) on maternal healthcare service use and providers were followed to generate the CoC variable. All recommendations, except the number of ANC visits, remain in the current WHO guidelines. These were, during the course of a pregnancy, each woman should have the following: i) receive at least four skilled ANC visits (this was changed to eight ANC visits in 2016, however, four skilled ANC visits is the current Bangladesh government’s recommendation); ii) be assisted by SBA during delivery; and iii) receive at least one PNC visit (for women and their newborns) within 24 hours of delivery from skilled healthcare personnel [32]. The variable was classified (see Table 2) as: high (women received each of the three recommended services (four or more ANC visits, birth assisted by SBA, and received at least one PNC visit within 24 hours of birth); moderate (women received at least two of the three recommended services), and low/none (women were not assisted by SBA, received no PNC, and had three or fewer ANC visits). We included women with no use of healthcare services as low/none level of CoC due to low response (20/4493).

### Explanatory variables

Women’s intention at conception of their most recent pregnancy that ended with a live birth was taken as the major exposure variable. Eligible women were asked i) “When you got pregnant with (name of last child that occurred within 3 years of survey date), did you want to get pregnant at this time?”. The response option was either “Yes” or “No”. If women responded “No”, they were asked, “Did you want to have a baby later on or did you not want any (more) children?”. Responses were then categorized as wanted (if the response was “yes” to the first question), mistimed (if the response was “later” to the follow-up question), and unwanted (if the response was “no more” in the follow-up question). The general validity of this measure of data collection and classification at the aggregate level has been repeatedly demonstrated in different settings, including Asian and African countries [33–38].

We adjusted the association between level of CoC and women’s intention at conception of their most recent pregnancy with a range of individual-, household-, and community-level factors. The factors were selected by using forward regression analysis on the list of factors found in our literature search [12,18–20,22,39]. The individual-level factors included were women’s age at the birth of their last child, decision-making about women’s healthcare, and women’s education. Partner’s education, partner’s occupation, exposure to mass media, and household wealth quintile were included as household-level factors. The BDHS’ reported variables, as well as the authors’ generated variables (based on individuals’ responses at the cluster level), were included as community-level factors. The BDHS’ reported factors were respondents’ place of residence and region at the time the survey was conducted. The authors’ generated factors were community-level illiteracy, community-level non-use of four or more ANC visits, community-level non-use of professional delivery care, and community-level non-use of PNC within 24 hours of birth. The details of the procedure to make these variables at the cluster level have been published elsewhere [27,30].

### Statistical analysis

The study was designed and reported in accordance with Strengthening the Reporting of Observational Studies in Epidemiology (STROBE) guidelines [40]. Basic sociodemographic characteristics of the women included were presented with means (±SD; for continuous variables) and frequency (for continuous and categorical variables). The different combinations of maternal healthcare services within the continuum of maternal healthcare was presented by percentages; the same was done separately for total pregnancies and across intention of pregnancy at conception. Dropout across the CoC (ANC through to PNC) was also calculated. Unadjusted and adjusted multilevel multinomial logistic regression models were carried out to assess the association between pregnancy intention and level of CoC. The unadjusted model was carried out to determine the raw effect of women’s pregnancy intention at conception on level of CoC. The adjusted model included individual-, household-, and community-level adjustment factors to determine the net effect of women’s pregnancy intention at conception on level of CoC. These models were employed using Stata’s generalized linear latent and mixed models (GLLAMM) command developed by Rabe-Hesketh and colleagues in 2005 [41]. The benefit of using this model is it can analyse the clustering structure of BDHS’ data (individuals nested within a household and households nested within a cluster), whereas previous studies found a multilevel model was deemed appropriate [12,41]. Sampling weights were also applied. Results were reported as odd ratios (OR) with 95% confidence intervals (CI). All analyses were done using Stata software version 15.1 (Stata Corp, College Station, Texas, USA).

## Results

### Characteristics of the sample

Table 1 shows the weighted sociodemographic characteristics of the women included. Of the 4,493 participants, most were aged 20–34 years (M = 23.63, SD = ±5.69), possessed a secondary level of education (M = 6.31, SD = ±3.83), and had ≤2 children (M = 2.16, SD = ±1.41). Around 26% of women reported that their last pregnancy that ended with a live birth was unintended at conception, 15.1% reported it was mistimed, and 10.9% reported it was unwanted.

**Table 1:** Weighted descriptive characteristics of women who gave at least one live birth within 3 years prior to the date of the 2014 Bangladesh Demographic and Health Survey (N = 4,493).

Variables	Percent
**Exposure variable: pregnancy intention at conception**	
Wanted	74.1 (71.3–76.7)
Mistimed	15.1 (13.4–16.8)
Unwanted	10.9 (9.4–12.6)
**Sociodemographic and reproductive characteristics**	
** Women’s age (in years) at birth of last child**	
**Mean (±SD)**	**23.63 (±5.69)**
≤19	28.0 (26.3–29.7)
20–34	67.7 (65.9–69.4)
≥35	4.4 (3.4–5.2)
**Women’s education**	
**Mean years of schooling (±SD)**	**6.31 (±3.83)**
Illiterate	14.2 (12.3–16.3)
Primary^2^	28.0 (26.1–29.9)
Secondary^3^	47.7 (45.2–50.3)
Higher^4^	10.2 (9.0–11.6)
**Parity**	
**Mean number (±SD)**	**2.16 (±1.41)**
≤2	70.0 (67.8–72.2)
>2	30.0 (27.8–32.2)
**Partner’s education**	
**Mean years of schooling (±SD)**	**5.84 (±4.66)**
Illiterate	23.9 (21.5–26.4)
Primary^2^	30.0 (28.0–32.0)
Secondary^3^	31.8 (29.4–34.3)
Higher^4^	14.4 (13.1–15.9)
**Partner’s occupation**	
Agricultural worker	25.8 (23.0–28.8)
Physical lobourer^b^	44.0 (41.6–46.4)
Services	5.9 (5.0–7.0)
Business	61.6 (19.9–23.4)
Other	2.8 (2.0–3.8)
**Household wealth quintile**	
Poorest	21.7 (19.0–24.6)
Poorer	18.9 (17.3–20.7)
Middle	19.1 (17.1–21.2)
Richer	20.6 (18.6–22.9)
Richest	19.7 (17.2–22.4)
**Place of residence**	
Urban	26.1 (23.5–28.9)
Rural	73.9 (71.1–76.5)

Note: N = total number of women included in the study.

### Level of the continuum of maternal healthcare

Table 2 contains information on elements of CoC defined following the WHO’s guidelines. Around half of all women (47.4%) who had a live birth in the three years before the survey had the lowest level of CoC and only 12.5% of women had the highest level of CoC (met WHO’s recommended levels).

**Table 2:** Grouping of maternal healthcare indicators (ANC visits, delivery assisted by SBA, and PNC visits) into the level of continuum of care (highest, moderate, low/none), Bangladesh Demographic and Health Survey, 2014 (N = 4,493).

Delivery and postnatal care	Antenatal care	
	Fewer than 4 ANC visits (n = 3305; 100%)	4 or more ANC visits (n = 1188; 100%)
**Delivery by skilled birth attendant**		
Yes (n = 1,990; 100%)	1,137 (34.4%)	853 (71.8%)
No (n = 2,503; 100%)	2,168 (65.6%)	335 (28.2%)
**Postnatal care for** mother-newborn dyads within 24 hours of birth		
Yes (n = 1,275; 100%)	700 (21.2%)	575 (48.4%)
No (n = 3218, 100%)	2;605 (78.8%)	613 (51.6%)
**Prevalence of levels of healthcare-seeking behaviours**		
**Level of the continuum of maternal healthcare**	**Number**	**Percent (95% CI)**
Low/no CoC	2,129	47.4 (45.8–52.1)
Moderate CoC	1,801	40.1 (36.9–42.0)
Highest CoC (WHO’s recommended level)	564	12.5 (10.1–13.4)

*Classifications reflect current World Health Organization guidelines; N = total number of women included in the study; n = number of women who used a particular service(s); percentage in the parentheses is column total.

Table 3 presents different combinations of maternal healthcare services received along the CoC by intention at conception of their last pregnancy. Of all the women, 27% had none of the maternal healthcare services, which increased to 37% for women who reported their last pregnancy was unwanted at conception. Around 14% of women with a wanted pregnancy received all services, and only 5.6% of women with an unwanted pregnancy received all services. SBA (3.7%) and PNC (0.5%) were uncommon without at least one ANC visit. After receiving at least one ANC visit, 7.2% of women had four or more ANC visits but no SBA and or PNC, 6.4% of women had four or more ANC visits and SBA but no PNC, and only 0.4% of women had PNC but did not have four or more ANC visits or SBA. These proportions were even lower among women who had a pregnancy which ended with a live birth that was mistimed or unwanted at conception.

**Table 3:** Proportion of women who received each combination of the continuum of maternal healthcare services by intention of pregnancy at the time of conception, Bangladesh Demographic and Health Survey, 2014 (N = 4493).

Continuum of maternal healthcare				Overall (n = 4,493)	Intention of pregnancy at conception		
ANC (1+)	ANC (4+)	SBA	PNC (women and newborns)		Wanted (n = 3,362)	Mistimed (n = 670)	Unwanted (n = 461)
				27.4	25.16	31.7	37.3
Yes				20.0	19.2	18.2	28.4
		Yes		3.7	3.6	3.9	4.2
			Yes	0.5	0.4	0.4	0.7
Yes	Yes			7.2	8.1	5.2	3.5
		Yes	Yes	3.2	3.1	3.9	3.5
Yes	Yes	Yes		6.4	7.0	6.0	2.8
Yes		Yes		6.9	7.3	6.1	4.8
Yes	Yes		Yes	0.24	0.26	0.2	0.2
Yes			Yes	0.4	0.4	0.6	0.0
Yes		Yes	Yes	11.5	11.6	12.5	9.1
Yes	Yes	Yes	Yes	12.5	13.8	10.7	5.6

Note: ANC (1+) = at least one ANC visit; ANC (4+) = at least 4 ANC visits; SBA = skilled birth attendance; PNC = postnatal care for women and newborns within 24 hours of delivery; N = total number of women included in the study; n = number of women with a particular type of pregnancy.

**Fig 2:**
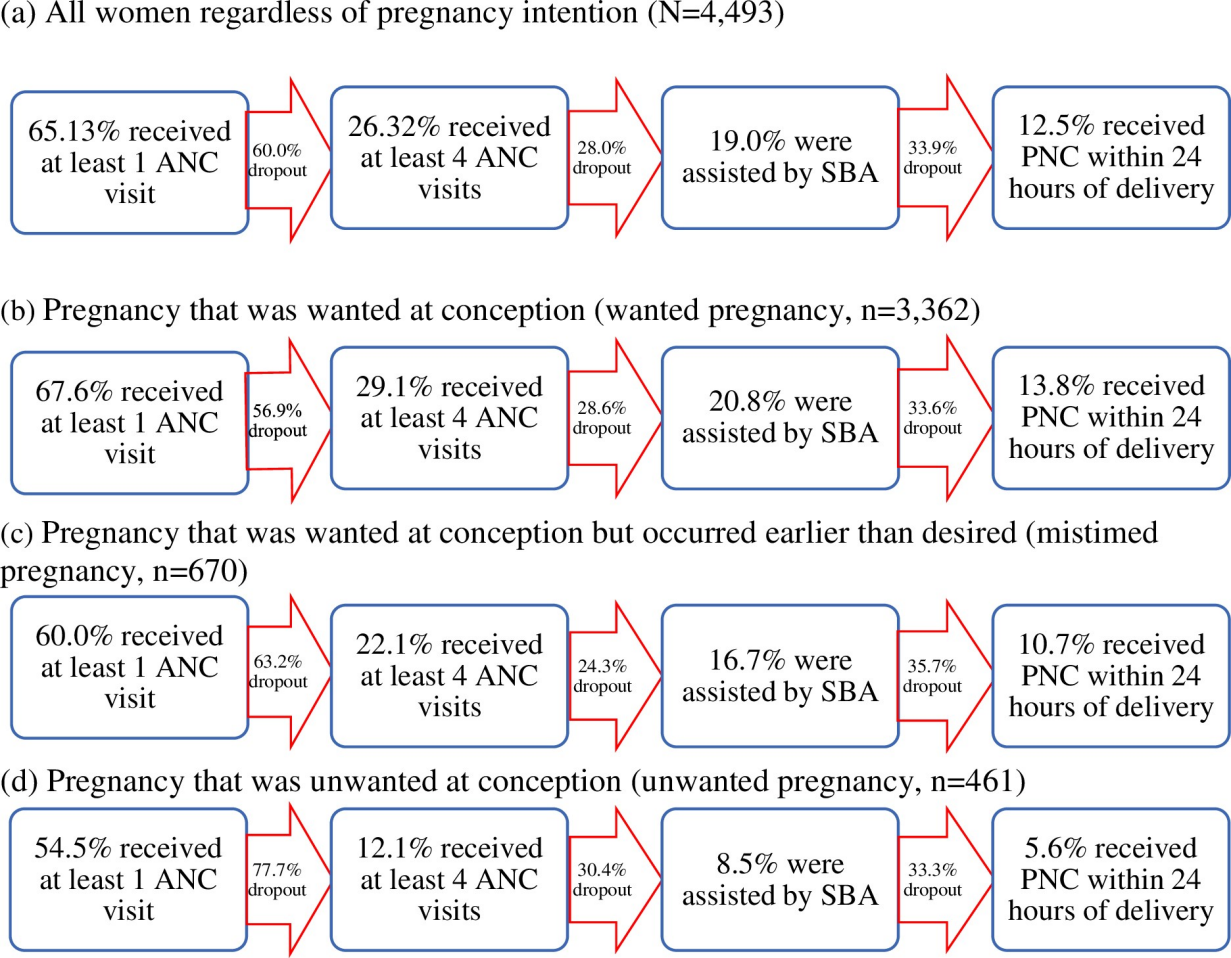
Women who discontinue the continuum of maternal healthcare through to PNC by women’s pregnancy intention at conception of their last pregnancy that ended with a live birth.

Fig 2 shows the changes in the proportion of women who attained CoC from one service to the next by pregnancy intention at conception. The proportion of dropout from one level to the next is also presented. Around 14% of women who had a wanted pregnancy at conception received all services, which declined from 68% use of at least one ANC. The proportion of women who received all services declined further if the pregnancy was mistimed or unwanted at conception. Only 5.6% of women who reported their last pregnancy was unwanted at conception received all services, which declined from 54.5% use of at least one ANC. The highest proportion of dropout in CoC occurred in the translation of one ANC visit to four or more ANC visits, followed by translation of SBA to PNC, and having four or more ANC visits to SBA. The trends associated with dropout were more pronounced for pregnancies that were unwanted or mistimed than those that were wanted at conception.

### Effect of pregnancy that was unintended at conception on level of continuum of maternal healthcare services use

Table 4 shows the unadjusted association between level of CoC and intention of pregnancy at conception. Likelihoods of using the moderate or highest level of CoC were found to be significantly lower among women who reported their last pregnancy was mistimed or unwanted at conception than women with a wanted pregnancy at conception. This association persisted following multivariate adjustment (see Table 5). In the fully adjusted model, compared to a wanted pregnancy, an unwanted pregnancy was found to be associated with 39% (OR 0.61, 95% CI 0.47–0.78) and 62% (OR 0.38, 95% CI 0.23–0.64) lower likelihoods of the moderate and highest levels of CoC, respectively. A pregnancy that was mistimed at conception was found to be associated with a 31% (OR 0.69, 95% CI 0.49–0.97) reduction in risk of achieving high CoC than a wanted pregnancy. However, the association between mistimed pregnancy and the moderate level of CoC was no longer statistically significant relative to low/no level.

**Table 4:** Multilevel multinomial logistic regression model with intention at conception of most recent pregnancy as the sole correlate of the level of continuum of maternal healthcare, Bangladesh Demographic and Health Survey, 2014.

	Level of continuum of maternal healthcare			
	Moderate vs. low/none level		Highest vs. low/none level	
	Odd ratio (95% CI)	*p-*value	Odd ratio (95% CI)	*p-*value
**Most recent pregnancy intention at conception: wanted conception (ref)**				
Mistimed conception	0.81 (0.64–1.00)	0.05	**0.67 (0.50–0.90)**	<0.01
Unwanted conception	**0.42 (0.33–0.54)**	<0.01	**0.25 (0.16–0.38)**	<0.01
Constant	1.10 (0.97–1.25)	0.12	0.37 (0.31–0.44)	<0.01
**Random effects**^**a**^				
Cluster-level variance (SE)^b^	1.51 (0.17)***			
Log-likelihood for fixed effects to random effects model	1114.94***			
Log-likelihood ratio test for the null model to the random effects model (chi-square)^c^	74.06***			

^a^We assume that the within cluster-level random effects are equal for the ‘moderate’ and ‘highest’ levels; therefore, only between cluster-level variance estimates are reported.

^b^Significance of random effects evaluated by comparing the model with a similar one in which random effects were constrained to zero.

^c^Compared to the null model with no-covariates.

***p<0.*01*.

**Table 5:** Odd ratios from multilevel multinomial logistic regression to assess the association between level of continuum of care and women’s pregnancy intention at conception, adjusting for individual-, household-, and community-level factors, Bangladesh Demographic and Health Survey, 2014.

	Level of continuum of maternal healthcare			
	Moderate level vs. low/none level		Highest vs. low/none level	
	Odd ratio (95% CI)	*p-*value	Odd ratio (95% CI)	*p-*value
**Most recent pregnancy intention at conception: wanted conception (ref)**				
Mistimed conception	0.85 (0.68–1.06)	0.15	**0.69 (0.49–0.97)**	<0.05
Unwanted conception	**0.61 (0.47–0.78)**	<0.01	**0.38 (0.23–0.64)**	<0.01
**Women’s age at birth of their last child: ≤19 years (ref)**				
20–34 years	0.92 (0.77–1.10)	0.34	1.00 (0.76–1.33)	0.99
≥35 years	1.18 (0.79–1.76)	0.43	1.73 (0.83–3.58)	0.14
**Decision-making about women’s healthcare: women involved (ref)**				
Women not involved	1.08 (0.93–1.26)	0.30	**1.30 (1.00–1.68)**	<0.05
**Women’s education: illiterate (ref)**				
Primary^2^	**1.40 (1.08–1.82)**	<0.01	**2.36 (1.14–4.86)**	<0.05
Secondary^3^	**1.84 (1.39–2.43)**	<0.01	**4.21 (2.04–8.71)**	<0.01
Higher^4^	**2.86 (1.87–4.35)**	<0.01	**9.85 (4.35–22.34)**	<0.01
**Partner’s education: illiterate (ref)**				
Primary^2^	1.13 (0.91–1.39)	0.27	0.94 (0.60–1.48)	0.79
Secondary^3^	1.38 (1.09–1.74)	<0.01	1.47 (0.93–2.32)	0.09
Higher^4^	2.24 (1.59–3.17)	<0.01	2.48 (1.43–4.30)	<0.01
**Partner’s occupation: agricultural worker**^**a**^ **(ref)**				
Labourer^b^	1.09 (0.89–1.32)	0.41	1.20 (0.82–1.76)	0.35
Services	1.16 (0.73–1.83)	0.53	1.74 (0.95–3.21)	0.08
Business	**1.35 (1.06–1.71)**	<0.01	**1.50 (1.00–2.28)**	<0.05
Other	1.29 (0.83–2.01)	0.26	1.02 (0.43–2.42)	0.96
**Exposure to mass media: not exposed (ref)**				
Moderately exposed	1.13 (0.94–1.36)	0.21	**2.28 (1.55–3.35)**	<0.01
Highly exposed	**1.43 (1.05–1.94)**	<0.05	**2.90 (1.77–4.74)**	<0.01
**Wealth status: poorest (ref)**				
Poorer	**1.49 (1.18–1.88)**	<0.01	1.53 (0.79–2.96)	0.21
Middle	**1.50 (1.17–1.92)**	<0.01	**2.10 (1.11–3.98)**	<0.05
Richer	**1.88 (1.41–2.51)**	<0.01	**3.34 (1.77–6.28)**	<0.01
Richest	**2.52 (1.81–3.51)**	<0.01	**5.19 (2.60–10.34)**	<0.01
**Place of residence: urban (ref)**				
Rural	1.10 (0.92–1.32)	0.32	1.04 (0.76–1.41)	0.82
**Region of residence: Barisal (ref)**				
Chittagong	0.91 (0.70–1.19)	0.49	0.94 (0.58–1.52)	0.80
Dhaka	0.93 (0.70–1.22)	0.59	1.19 (0.73–1.92)	0.48
Khulna	**1.37 (1.04–1.81)**	<0.05	1.41 (0.83–2.37)	0.20
Rajshahi	1.28 (0.96–1.70)	0.09	1.46 (0.88–2.42)	0.14
Rangpur	**1.41 (1.05–1.89)**	<0.05	1.56 (0.91–2.66)	0.11
Sylhet	0.90 (0.66–1.23)	0.51	1.31 (0.77–2.25)	0.32
**Community-level illiteracy: low (<25%, ref)**				
Moderate (25–50%)	1.14 (0.96–1.36)	0.15	0.98 (0.75–1.27)	0.86
High (50%)	0.96 (0.71–1.29)	0.76	0.97 (0.57–1.64)	0.91
**Community-level four or more antenatal care services non-use:** Low (0–49%; ref)				
High (50–100%)	**0.55 (0.43–0.70)**	<0.01	**0.26 (0.20–0.35)**	<0.01
Community-level professional delivery care services non-use^*^: Low (0–49%; ref)				
High (50–100%)	**0.26 (0.22–0.32)**	<0.01	**0.46 (0.32–0.64)**	<0.01
**Community-level not appropriate postnatal care visits:** Low (0–49%; ref)				
High (50–100%)	0.79 (0.59–1.05)	0.09	**0.22 (0.15–0.30)**	<0.01
**Random effects**^**a**^				
Cluster-level variance (SE)^b^	0.03 (0.04)***			
Log-likelihood for fixed effects to random effects model	609.44***			
Log-likelihood ratio test for the null model to random effects model (chi-square)^c^	1624.59***			

*Professional personnel to provide delivery care include qualified doctors, nurses, midwives, paramedics, family welfare visitors, and community skilled birth attendants. ^a^We assume that the within cluster-level random effects are equal for the ‘moderate’ and ‘highest’ levels; therefore, only between cluster-level variance estimates are reported.

^b^Significance of random effects evaluated by comparing the model with a similar one in which random effects were constrained to zero.

^c^Compared to the null model with no-covariates.

***p<0.*01*.

Of the different factors adjusted, women’s involvement in their healthcare decision-making and increased years of education were found to be associated with higher likelihoods of achieving the highest and the moderate levels of CoC than the lower level of these corresponding characteristics. Similarly, at the household-level, partner’s engagement in business (reference group was agricultural worker), higher exposure to mass media (reference group was not being exposed to mass media), and improved household wealth quintile (reference group was household poorest wealth quintile) were the factors that were found to be associated with increased likelihoods of achieving the highest and the moderate levels of CoC. Turning to the community-level factors, we found up to an 82% lower likelihood of achieving the highest level of CoC among women residing in a community where more than 50% of women did not use four or more ANC visits, SBA, or PNC visits than women residing in a community with at least 50% use of these services. These reduced odds were approximately similar for the moderate level of CoC.

## Discussion

This study provides a systematic analysis of CoC completion through to PNC, based on the most recent nationally representative household survey data in Bangladesh. Overall, we found that 12.5% of women had CoC across all three services. This figure declined by around half (5.6%) among women whose pregnancies were unwanted at conception. The highest dropout from CoC was found in the translation of at least one ANC visit to four or more ANC visits. We found these low levels of CoC, despite Bangladesh being acclaimed globally for its increase of ANC and SBA in accordance with the MDGs. Therefore, the current progress of maternal healthcare service use will minimally contribute to the SDG targets of universal healthcare coverage for optimizing maternal and newborn adverse health outcomes. Importantly, this challenge of improving CoC cannot be overcome with the current supply-oriented and disjointed healthcare provision in Bangladesh. Moreover, our study indicates that a high occurrence of pregnancies that are unintended at conception (at around 26%) is a barrier to CoC and therefore will further challenge Bangladesh’s aim of achieving its SDG targets. The findings suggest a need for policies to integrate healthcare services with a particular focus on women who have pregnancies that are unintended at conception.

This study found a dropout rate of around 80% for women who started with one ANC visit (65%) to completion of CoC (12.5%). This dropout rate was higher than in Pakistan (72.6% dropout, 12% CoC) [18], the average of three South Asian countries (Bangladesh, Nepal, Pakistan; 68.67% dropout, 24.5% CoC) [12], and in Cambodia (50% dropout, 60% CoC) [19]. However, Bangladesh had a lower dropout rate than several countries, including the average of six sub-Saharan African countries (Ethiopia, Malawi, Rwanda, Senegal, Tanzania, and Uganda; 83.9% dropout, 13.9% CoC) [12], Ghana (92% dropout, 8% CoC) [20], and Lao PDR (91.34% dropout, 6.8% CoC) [39]. Importantly, such differences of CoC between Bangladesh and other countries are reflective of the differences in uptake of individual maternal healthcare services use, including ANC, SBA, and PNC. These services are always found to be lower in Bangladesh than in other Asian countries (e.g. Pakistan, Nepal, and Cambodia) [19,42,43], however, higher than African countries [12]. Further increases in the dropout of completion of CoC were found among women who had unwanted (62%) and mistimed (31%) pregnancies at conception than wanted pregnancies. Previous studies in Bangladesh found likelihoods of using at least four ANC visits, SBA, and first PNC visit within 24 hours of delivery declined by around 33%, 35%, and 42%, respectively, among unwanted pregnancies than wanted pregnancies [27,29,30].

The results point to several pathways through which pregnancy intention can influence CoC completion; however, the majority involve healthcare system level factors, individual and household level factors (such as lower education and household wealth quintile) associated with unintended pregnancy, and women’s characteristics following unintended conception. In its current health care structure in Bangladesh, family planning services and maternal healthcare services are provided separately through the Directorate General of Family Planning (DGFP) and the Directorate General of Health Services (DGHS), respectively, along with focus on supplying these services rather than considering demand-side factors (such as behavioural change of people in the community) [44,45]. Inadequate or no counselling by family planning providers (which plays a role in the number of unintended pregnancies) and their current minimal (or lack of) coordination with maternal healthcare service providers could, therefore, contribute to lower CoC among women with unintended conceptions. Moreover, the occurrence of unintended conceptions are usually found to be higher among disadvantaged women in terms of individual- and household-level factors and women residing in disadvantaged communities (including those with lower education and higher poverty) [46,47]. These factors have been found to be independently associated with the lower use of ANC, SBA, and PNC in Bangladesh and LMICs [48–50]. Post-conception characteristics, including household-level response (family members and partner opposition) and the psychological consequences following an unintended pregnancy (depression and anxiety) are other possible factors impacting on CoC among women with unintended pregnancies [21]. Accessing all components of CoC requires multiple visits to healthcare facilities which requires additional travel money and more assistance with visiting healthcare facilities. Previous studies in Bangladesh [27,29,30] and LMICs [21,51] reported these as significant deterrents to the use of ANC, SBA, and PNC following the occurrence of an unintended pregnancy. Late pregnancy detection and decisions around pregnancy termination may be other important reasons for lower CoC among women with an unintended pregnancy [21]. As these women have fewer ANC visits, there are also missed opportunities for healthcare providers to motivate them to access SBA and PNC [30]. Consequently, the progress in CoC in general, and among unintended pregnancies in particular, requires policies that address issues related to the country’s healthcare system (e.g. the disjointed approach to providing healthcare), women’s place within society, as well as sociodemographic and post-conception characteristics.

The Alma-Ata Declaration of Health in 1978 by the WHO has emerged as a major milestone in Bangladeshi healthcare policy to ensure primary healthcare at the grass-roots level [52]. Therefore, the focus has always been given to making a pluralistic healthcare system [53], developing community-based approaches by ensuring partnerships with non-governmental organizations [54], the establishment of community clinics [27], and rounds of sector-wise health and nutritional programs [55]. Consequently, significant improvements have been reported in the provision of reproductive and maternal healthcare services [56] and family planning services [54]. However, what is still lacking are policies to ensure the continuity of using family planning services to PNC services through integrated healthcare services. For instance, many women use family planning services, however, drop out without accessing maternal healthcare services [57,58]. Importantly, a significant number of women use maternal healthcare services when required because of complications (need-based) rather than access all services for better pregnancy outcomes (merit-based) [57,58]. Moreover, a significant proportion of women who start ANC services drop out later from using the recommended number of ANC, SBA, and PNC services [57]. Therefore, integration of family planning services with maternal healthcare services and among several streams of maternal healthcare services along with strong monitoring for continuity could be effective options to reduce this dropout after starting access of services. However, such integration and monitoring for continuity in Bangladesh requires several challenges to be addressed at the national level.

First, there is an acute shortage of human resources in healthcare sectors in Bangladesh (with around 800,000 workers), which has existed for decades [59], and poor sustainability and retention of community health workers (current dropout rate is 12–44%) [60,61]. Ensuring health workers in the primary level stay is therefore a challenge for the coordination of services at home and in the community and monitoring for continuity. Previous studies in Bangladesh [62] and Tanzania [63] found these are significant for initiating reproductive healthcare services, as well as continuing services use through to PNC. Second, there is an inadequate focus on the quality of services given, which is now recognized as an important deterrent to CoC [64,65]. This might be a reason for the high dropout after having at least one ANC visit that was found in this study. Third, ignorance of the demand-side factors in healthcare policies, including community support systems, mobilization of community participation, and people’s behavioural change [54,62]. Establishing public healthcare facilities through supply-oriented healthcare policies, even at the community-level, would therefore have little effect on CoC completion. The roots for this could be the conservative cultural environment, cultural norms, and traditional beliefs in Bangladesh [66,67]. For instance, the patriarchal system in Bangladesh often restricts women’s decision-making capability and the economic affordability of visiting healthcare facilities. Besides, cultural norms within Islam (the religion of more than 90% of the total population in Bangladesh) do not permit women to leave their homes while pregnant, with pregnant women believed to be highly vulnerable to evil spirits (one reason why their mobility is restricted, particularly during delivery and soon after childbirth) [68]. These practices are even more prevalent among women with disadvantaged individual-, household-, and community-level characteristics (e.g. lower education, socioeconomic status) [27,67,69]. These factors are also associated with lower contraception use (which might be a major factor for the higher occurrence of unintended pregnancy) [28] and even more restriction of women’s movement during pregnancy, predominantly in early pregnancy and in the early postpartum period [70,71]. Essentially, these are the stages where this study found higher dropout from CoC, findings that are consistent with the literature on LMICs [12,20,22,24,27,31,39]. Therefore, further progress in achieving CoC for Bangladeshi women, particularly among women with unintended pregnancies, requires the availability of healthcare services, consideration of behavioural change of people in the community, and meeting the current shortage of workforce.

Moreover, such supply-oriented policies and disjointed healthcare services in Bangladesh make maternal healthcare services mainly visit-based (women and newborns visit health facilities to receive services), even for healthcare facilities available at the community-level. However, previous studies in Bangladesh have found that in order to increase the use of maternal, newborn and child health, a visit-based approach is less effective than a services-based approach (healthcare providers visit women’s homes and provide services there) [62,66]. Similarly, a study in Ghana found higher CoC with a services-based approach (10.3%) rather than a visit-based approach (7.9%) [22]. Integration of family planning workers with mainstream maternal healthcare services could help Bangladesh to provide a services-based like maternal healthcare service. For instance, in an integrated service, family planning workers would be given additional responsibility for educating and monitoring maternal healthcare service use along with their current responsibility of family planning and distributing contraception. Currently, there is no governmental effort at the field level (in which family planning operates) to ensure maternal healthcare services use or monitoring of service use [31]. Therefore, this integration could contribute to increasing average maternal healthcare services use along with continuity. It also reduces the differences in maternal healthcare services use that occur because of the sociodemographic characteristics of women and their partners along with demand-side factors (being disadvantaged in any of these characteristics is found to be associated with a higher occurrence of unintended pregnancy) [46,47]. Therefore, family planning workers’ responsibility to focus on these groups and ensure proper education about the importance of maternal healthcare services use and monitoring for continuity is important for improving CoC in the country.

There were several limitations to our study. First, as it was a cross-sectional study, the relationship between pregnancy intention at conception and CoC is correlational only. Second, all components of CoC and pregnancy intention data were collected based on women’s retrospective (recall) responses, which may be subject to reporting errors (e.g. recalling the exact number and timing of healthcare services use). Moreover, women’s feelings about pregnancy can change in many ways over a 3 year period, which could lead to considerable ambivalence in reporting pregnancy status [37]. For instance, a woman’s opinion about pregnancy and timing might change after the birth of her child; therefore, intentional pregnancies may be reported as “unwanted” or “unintended”, and unintentional pregnancies may be reported as “wanted” [37]. An intended pregnancy could also be reported as “unintended” (or “unwanted”) if the outcome of the pregnancy (the child’s sex) is the opposite of the parents’ desire for a child of a particular sex. For instance, an Indian study reported a higher likelihood of unintended pregnancy among women whose child was female than male [72], though, no study in LMICs reported the age-specific changes of unintended pregnancy reporting. This could be because of parents’ higher preference of a male than a female child- a context which is also common in Bangladesh [11]. Moreover, retrospective responses make distinguishing between mistimed and unwanted conception challenging because of the potential nuance of stating a pregnancy is mistimed or unwanted following upto 3 years of conception occurred due to elapsed time. Third, we did not have data on the distance of the healthcare facilities from the women’s homes or the quality of services, which are important for CoC in visit-based services in Bangladesh. Moreover, the timing of pregnancy detection and the first ANC visit are important for CoC although they were not adjusted for because of lack of data. Moreover, as we weighted the forms of maternal healthcare services equally, it is important to acknowledge that the risk factors for dropout at each stage may differ. However, previous studies of this kind in Bangladesh found around consistent results in respect to the determinants of maternal healthcare services use with the current study [27,29,30]. Despite these limitations, the current study is the first in Bangladesh and LMICs that assesses the effect of unintended pregnancy at conception on CoC completion based on data from a unique representative survey. Use of the CoC framework, appropriate statistical adjustment for the survey design, and adjustment of a comprehensive range of factors enabled us to make the strongest recommendations for future policies in efforts to improve maternal health.

## Conclusion

This study highlighted the importance of an integrated approach to maternal healthcare services, from pregnancy to the early post-partum stage. Findings suggest only 12.5% of women had a CoC in their last pregnancy, which decreased by around half (5.6%) among women who had a pregnancy that was unwanted at conception. Higher dropout from the CoC was reported in translating at least one to four or more ANC visits. Following adjustment for a comprehensive range of individual-, household-, and community-level factors, unintended pregnancy at conception was found to be strongly associated with the level of CoC attained. Different individual-, household-, and community-level factors, including lower women’s education, lower household socio-economic status, and lower use of ANC and delivery care services at the community level were also found to be influential determinants of lowering CoC. To ensure CoC, particularly among women who have unintended conceptions, policies are required to enhance healthcare services at the community level, along with the focus on demand- and supply-side interventions together. It is also important for policies to integrate family planning and maternal healthcare services.

## Abbreviations

CoC: Continuum of care
LMICs: Low- and Middle-Income Countries
ANC: Antenatal care; SBA: Skilled birth attendance
PNC: Postnatal care
SDG: Sustainable Development Goal
MDGs: Millennium Development Goals
BDHS: Bangladesh Demographic and Health Survey
USAID: United States Agency for International Development
UNICEF: The United Nations Children’s Fund
OR: Odds Ratio
