# Correction: Assessing the effect of pregnancy intention at conception on the continuum of care in maternal healthcare services use in Bangladesh: Evidence from a nationally representative cross-sectional survey

**DOI:** 10.1371/journal.pone.0244264

**Published:** 2020-12-15

**Authors:** Md Nuruzzaman Khan, Melissa L. Harris, Deborah Loxton

An additional affiliation is missing for the first author. Md Nuruzzaman Khan is also affiliated with #2: Faculty of Health and Medicine, School of Medicine and Public Health, University of Newcastle, Newcastle, Australia
