# Supplementary material for: Assessing the effect of pregnancy intention at conception on the continuum of care in maternal healthcare services use in Bangladesh: Evidence from a nationally representative cross-sectional survey
Source: PLoS One. 2020 Nov 20;15(11):e0242729. doi: 10.1371/journal.pone.0242729 (PMC7678970; doi:10.1371/journal.pone.0242729)
Supplement: Attachment — Submitted filename: Response to Reviewers.docx [file pone.0242729.s001.docx]

| **Comments and suggested changes** | **Changes made and authors’ reply** |
| --- | --- |
| **Reviewer#1** |  |
| This paper investigates the association between having an unintended birth and maternal use of healthcare – specifically continuity of care during and after pregnancy – among women in Bangladesh. The strengths of the paper lie in the large sample, completeness of the measure of unintended births (unwanted and mistimed), and the clear writing. I also want to commend the authors on Figure 1, which provides a very nice depiction of the percentages of women who completed CoC or dropped out. Despite these strengths, some areas of the paper would benefit from additional revision. I include several questions and suggestions below in the aim of strengthening the paper. | We have edited the manuscript in line with your suggestions and questions. The itemized responses are provided below. |
| One limitation of the current study is that the authors rely solely on retrospective data. This needs to be clarified when describing the data (pg. 8). Additionally, there is a robust literature on the biases inherent in retrospective measures that should be considered in the discussion section (pg. 17) to ascertain what type of bias may have arisen due to this measurement strategy. Many studies from a variety of global settings have found that shifts in women’s reports of intentions are predictable – and tied to intendedness - rather than random as is suggested in the paper. Another potential source of bias is selection into unintended pregnancy. How might this influence the results? Other studies have used analytic strategies like propensity score weighting to control for selection into unintended pregnancy. Although I do not believe that is necessarily required here, the authors should at least acknowledge this source of bias and discuss how it might influence their results. | We have edited the second sentence in the *Data* sub-section under the Methods to mention that the data analyzed in this study were retrospective in nature. The edited sentence now reads *(See page 8 at the revised manuscript for details)-*  *The conditions of inclusion were as follows: i) provided a response to the retrospective question regarding giving live birth in the three years prior to the date of the survey; ii) responded to the questions on the use of maternal healthcare services during their pregnancy (most recent pregnancy if more than one pregnancy occurred); and iii) reported on pregnancy intention at conception of the included pregnancy (which was reported retrospectively).*    We have also edited the limitations section of the Discussion (pg. 17) to clarify the use of retrospective data in the present study. This section now reads *(See last paragraph at page 19 in the revised manuscript for details)-*  *There were several limitations to our study. First, as it was a cross-sectional study, the relationship between pregnancy* *intention at conception and CoC is correlational only. Second, all components of CoC and pregnancy intention data were collected based on women’s retrospective (recall) responses, which may be subject to reporting errors (e.g. recalling the exact number and timing of healthcare services use). Moreover, women’s feelings about pregnancy can change in many ways over a 3 year period, which could lead to considerable ambivalence in reporting pregnancy status [71]. For instance, a woman’s opinion about pregnancy and timing might change after the birth of her child; therefore, intentional pregnancies may be reported as “unwanted” or “unintended”, and unintentional pregnancies may be reported as “wanted” [71]. An intended pregnancy could also be reported as “unintended” (or “unwanted”) if the outcome of the pregnancy (the child’s sex) is the opposite of the parents’ desire for a child of a particular sex. Moreover, retrospective* *responses make distinguishing between mistimed and unwanted conception challenging as many women might be unable to determine whether an unintended pregnancy was mistimed or unwanted. However, any such bias is likely to be random.* |
| In the Outcome Variable section, it is important to include the actual survey questions used to create the CoC measure – similar to what is done in the Explanatory variables section (see pg. 8). This would clear up several of my questions about the items used to construct this measure, such as how a skilled birth attendant is defined | We have added the survey questions for the variables used to create the CoC measure. This reads *(See last paragraph at page 8 in the revised manuscript for details)-*  *The outcome variable, CoC, was generated from women’s responses to questions on the use or non-use of ANC, SBA, and PNC. Two survey questions regarding ANC services were used to determine if women had received ANC, and if so, how many times. Each eligible woman was first asked, “Did you see anyone for antenatal care for this pregnancy?”. Women who responded “Yes” to this item were then asked, “How many times did you receive antenatal care during this pregnancy?”. SBA status was derived by combining women’s* *responses to the two questions that were asked to determine who had provided care during delivery (“Who assisted with the delivery of [name of the most recent child born in the past three years])?” and where delivery had occurred (“Where did you give birth to [name of the most recent child born in the past three years])?”. Four additional questions collected information on the timing of the first PNC visit for women and newborns, as well as providers of PNC. Each eligible woman was first asked, “Did anyone check on you and your baby’s health following birth?” Further questions were asked to determine the timing (“How long after delivery did the first check take place?”), place (“Where did this first check take place?”), and providers (“Who checked on your health at that time?”) of PNC. These items were recoded to generate the CoC variable depending upon the continuity of using maternal healthcare through to PNC. The WHO guidelines in 2014 (which were in use at the time of survey completion) on maternal healthcare service use and providers were followed to generate the CoC variable. All recommendations, except the number of ANC visits, remain in the current WHO guidelines. These were, during the course of a pregnancy, each woman should have the following: i) receive at least four skilled ANC visits (this was changed to eight ANC visits in 2016, however, four skilled ANC visits is the current Bangladesh government’s recommendation); ii) be assisted by SBA during delivery; and iii) receive at least one PNC visit (for women and their newborns) within 24 hours of delivery from skilled healthcare personnel [32].* |
| In the literature review, the unintended fertility rate in Bangladesh is described as being 46% of all pregnancies (pg. 6), but the current study finds that only 26% of births are unintended. Please include a discussion of why you believe there is such a large difference between these percentages (selection? Measuring pregnancies vs. births?), and describe how this may have affected your results and therefore your conclusions. | Around 43% of the total pregnancies in Bangladesh are unintended at conception, before considering abortion. We have now edited the relevant sentence in the Introduction section (pg. 6) to clarify this issue. This reads *(See last paragraph at page 6 in the revised manuscript for details)-*  *Obstetric complications are even higher for women with an unintended pregnancy, who represent around 43% of total pregnancies at conception in Bangladesh (including pregnancies that are terminated) and around one-quarter of the total pregnancies that ended with live births [26-28].*  Since the analysis of this study was limited to women who have had an unintended pregnancy that ended with a live birth (i.e. excluding women who terminated pregnancies) and the outcome variables were maternal healthcare services that women use across the course of continued pregnancy, therefore such differences in the percentage did not affect this study results and conclusion. Focusing on abortion services following an unintended pregnancy is beyond the scope of the current study. |
| The authors claim that they are able to make the “strongest recommendations for future policies” (pg. 18), but from my reading, many of the policy recommendations are rather vague. This is the area of the discussion that needs the most attention. For example, one of the authors’ recommendations is to create policies that “integrate healthcare services” (pg. 14). Can the authors provide some concrete suggestions on how Bangladesh – or LMICs more broadly – might do this and/or what these policies might look like? Similar questions apply to the recommendation for policies to “consider a range of factors…” (pg. 15). Addressing these questions and providing concrete policy recommendations would greatly strengthen the paper and its potential to contribute to positive changes in maternal health services and utilization in Bangladesh. | We have now edited the relevant paragraphs 3-6 in the Discussion section to address these issues. Please see the relevant sections (at pages 15-19) for details. |
| One of the study’s findings is that Bangladesh falls in the middle in terms of its dropout rate. I’d like to see the authors offer some explanations of why they think this is the case. Also, what could Bangladesh do better to emulate the South Asian countries mentioned on page 14? | We have now added sentences to explain why Bangladesh falls in the middle among Asian and African countries in terms of the dropout from CoC. These read *(See first paragraph at page 15 in the revised manuscript for details)-*  *Importantly, such differences of CoC between Bangladesh and other countries are reflective of the differences in uptake of individual maternal healthcare services use, including ANC, SBA, and PNC. These services are always found to be lower in Bangladesh than in other Asian countries (e.g. Pakistan, Nepal, and Cambodia) [19, 41, 42], however, higher than African countries [12].*  We have also presented a broad description of potential policies that could help Bangladesh to emulate maternal healthcare services than Asian countries in the discussion. |
| Cultural factors are mentioned as a barrier to increasing CoC among women in Bangladesh. How might these be addressed in policy creation and implementation? Addressing this would strengthen the discussion on page 16. | We have edited the relevant section in the discussion to include a description of cultural issues in Bangladesh by mentioning policies to address effectively these issues to increase maternal healthcare services. This reads *(See first paragraph at page 18 in the revised manuscript for details)-*  *The roots for this could be the conservative cultural environment, cultural norms, and traditional beliefs in Bangladesh [65, 66]. For instance, the patriarchal system in Bangladesh often restricts women’s decision-making capability and the economic affordability of visiting healthcare facilities. Besides, cultural norms within Islam (the religion of more than 90% of the total population in Bangladesh) do not permit women to leave their homes while pregnant, with pregnant women believed to be highly vulnerable to evil spirits (one reason why their mobility is restricted, particularly during delivery and soon after childbirth) [67]. These practices are even more prevalent among women with disadvantaged individual-, household-, and community-level characteristics (e.g. lower education,* *socioeconomic status) [27, 66, 68]. These factors are also associated with lower contraception use (which might be a major factor for the higher occurrence of unintended pregnancy) [28], and even more restriction of women’s movement during pregnancy, predominantly in early pregnancy and in the early postpartum period [69, 70]. Essentially, these are the stages where this study found higher dropout from CoC, findings that are consistent with the literature on LLMICs [12, 20, 22, 24, 27, 31, 38]. Therefore, further progress in achieving CoC for Bangladeshi women, particularly among women with unintended pregnancies, requires the availability of healthcare services, consideration of behavioural change of people in the community, and meeting the current shortage of workforce.* |
| **Reviewer#2** |  |
| This study investigates an important issue – continuum of pregnancy-related care (CoC) in low and lower-middle-income countries and the impact that unintended pregnancy has on receipt of care. This work is particularly important for recognizing the potential disparities and opportunities for intervention in improving receipt of recommended maternal health services, particularly among women who experience unintended pregnancy. Below are additional comments that hopefully will improve the contribution that this article can make, mostly in terms of the presentation and framing of the dependent variable and reporting results. Overall, this is a thoughtful article, which extends the current body of maternity care research beyond simple measures of ANC, SBA and PNC. |  |
| **Abstract** |  |
| 1. Methods: It would be helpful if the authors could note the data source (DHS Bangladesh 2014) in the abstract, so readers understand the type of dataset used for the analysis. | As suggested, we have added the following sentence in the Methods section of the Abstract.  *Data from 4,493 mother-newborn dyads who participated in the cross-sectional 2014 Bangladesh Demographic and Health Survey were analysed.* |
| 2. Results: The language about “maintaining” vs. “achieving” CoC is confusing. Per the definition of the CoC (high, moderate, and low/none), the measure is complete when all components of pregnancy-related care are considered. Use of the word “maintain” here makes it seem as though the study examines CoC as a measure over time, instead of within the context of a single index pregnancy. Suggest to reword any use of “maintain” as “achieve/attain” throughout the abstract and manuscript for clarity. | We have now addressed this issue in the results of the abstract and throughout the manuscript. |
| **Methods** |  |
| **Data** |  |
| The subsample of women included in this analysis must have had a livebirth in three years prior to the survey; responded to the question on maternity care, and reported retrospective pregnancy intention. What proportion of women aged 15-49 had a birth in the prior three years, but did not have complete measures on all three components of CoC? It might be interesting, or at least provide some insight, if the authors could note which components of those eligibility criteria resulted in exclusion (i.e. some kind of flowchart with numbers of how they reached the final sample, for reference). Unclear if the jump from 17,863 to 4,493 women was due to a relatively low number of women who had a birth within the three years prior; incomplete data on maternal health services; or no pregnancy intention reported at conception, which are relevant for understanding the broader study sample from which this analytic sample was derived. | We have now added Figure 1 and presented details of the survey sample selection procedure and for this study. |
| **Outcome variable** |  |
| The authors indicate that they followed the WHO’s 2014 guidelines for maternity care, including receipt of at least four ANC visits. They also note that this changed to eight in 2016. Why did the authors choose to assess 4+ visits instead of 8+ visits, given the change in recommendations? Were any sensitivity analyses conducted to see how this altered the distribution of outcomes? I suspect far fewer women would have achieved a “high” level of continuum of care, but might provide an interesting comparison at least to address in the discussion. | Receiving at least four ANC visits during pregnancy is still the Bangladesh government guideline. We have clarified this issue in the relevant sentence *(See first paragraph at page 9 in the revised manuscript for details).*  *The WHO guidelines in 2014 (which were in use at the time of survey completion) on maternal healthcare service use and providers were followed to generate the CoC variable. All recommendations, except the number of ANC visits, remain in the current WHO guidelines. These were, during the course of a pregnancy, each woman should have the following: i) receive at least four skilled ANC visits (this was changed to eight ANC visits in 2016, however, four skilled ANC visits is the current Bangladesh government’s recommendation); ii) be assisted by SBA during delivery; and iii) receive at least one PNC visit (for women and their newborns) within 24 hours of delivery from skilled healthcare personnel [32].*  Moreover, as only 293 of the 4493 women analysed, reported 8+ ANC visits, this number would further reduce the numbers of SBA and PNC. Therefore, it was not possible to consider the new WHO guidelines for this study. |
| While the authors note that their development of the outcome variable aligns with the WHO’s guidance, I would like to know more about their decision to “weight” all three components of care equally. For example, is a woman who received ANC and PNC, but no SBA likely to encounter different health risks than women who received a different combination of care (e.g., ANC, SBA, no PNC)? Do the authors anticipate that this would have altered the outcome? | We weighted all three components of care equally to develop the outcome variable (CoC). We have outlined the limitations of this approach in the strength and limitations section of the discussion and tables’ titles. The relevant edited sentence in the study’s strength and limitations section now reads *(See first paragraph at page 19 in the revised manuscript for details)-*  *Moreover, as we weighted the forms of maternal healthcare services equally, it is important to acknowledge that the risk factors for dropout at each stage may differ. However, previous studies of this kind in Bangladesh found around consistent results in respect to the determinants of maternal healthcare services use with the current study [27, 29, 30]. Despite these limitations, the current study is the first in* *Bangladesh and LLMICs that assesses the effect of unintended pregnancy at conception on CoC completion based on data from a unique representative survey. Use of the CoC framework, appropriate statistical adjustment for the survey design, and adjustment of a comprehensive range of factors enabled us to make the strongest recommendations for future policies in efforts to improve maternal health.* |
| Throughout the paper, the authors use the terminology “dropout” to describe women who received early components of CoC, but did not receive later components. This language, which reframes the dependent variable to consider each “stage” of the CoC, should be described in this part of the methods section. As a reviewer, it was not clear in the methods that the analysis would include more nuanced assessments of the CoC, other than the three-level categorical variable that is described. | We have edited this section and added sentences to clarify this issue. This now reads *(See first paragraph at page 9 in the revised manuscript for details)-*  *These items were recoded to generate the CoC variable depending upon the continuity of using maternal healthcare through to PNC. The WHO guidelines in 2014 (which were in use at the time of survey completion) on maternal healthcare service use and providers were followed to generate the CoC variable. All recommendations, except the number of ANC visits, remain in the current WHO guidelines. These were, during the course of a pregnancy, each woman should have the following: i) receive at least four skilled ANC visits (this was changed to eight ANC visits in 2016, however, four skilled ANC visits is the current Bangladesh government’s recommendation); ii) be assisted by SBA during delivery; and iii) receive at least one PNC visit (for women and their newborns) within 24 hours of delivery from skilled healthcare personnel [32]. The variable was classified (see Table 2) as: high (women received each of the three recommended services (four or more ANC visits, birth assisted by SBA, and received at least one PNC visit within 24 hours of birth); moderate (women received at least two of the three recommended services), and low/none (women were not assisted by SBA, received no PNC, and had three or fewer ANC visits). We included women with no use of healthcare services a*s low/none level of CoC due to low response (20/4493). |
| **Explanatory variable** |  |
| It would be helpful if the authors clearly stated that “women’s intention at conception” was the woman’s fertility/pregnancy intention. The term “intention at conception” is a bit abstract; “index pregnancy intention” may be a better term for this concept throughout the manuscript. | As suggested, we have edited the relevant sentences throughout the manuscript and replaced the term “*women’s intention at conception*” by “*women’s intention of pregnancy at conception*”. |
| Authors should also indicate that this is a retrospectively reported measure, one that may be reporting on a pregnancy from up to three years prior. Limitations of such a measure should be noted. | We have now added sentences to clarify this issue. The edited explanatory variable section now reads *(See last paragraph at page 9 in the revised manuscript for details)-*  *Women’s intention at conception of their most recent pregnancy that ended with a live birth was taken as the major exposure variable. Eligible women were asked i) “When you got pregnant with (name of last child that occurred within 3 years of survey date), did you want to get pregnant at this time?”. The response option was either “Yes” or “No”. If women responded “No”, they were asked, “Did you want to have a baby later on or did you not want any (more) children?”. Responses were then categorized as wanted (if the response was “yes” to the first question), mistimed (if the response was “later” to the follow-up question), and unwanted (if the response was “no more” in the follow-up question). Importantly, these questions were asked together with contraception use or non-use at that time. Therefore, potential* *ambivalence associated with a retrospective approach to data collection was reduced greatly as women were able to classify more accurately whether this pregnancy was due to not using contraception because of hoping for a child, contraception failure, or lack of access or use of contraception. The general validity of this measure of data collection and classification at the aggregate level has been repeatedly demonstrated in different settings, including Asian and African countries [33-37].*  We have also added limitations of such retrospective approach of measuring unintended pregnancy in the limitation section under the discussion. Please see the limitations section of the manuscript and our response to reviewer 1’s comment on limitation (second comment in the column). |
| Were women who responded “Don’t know” to the question of “whether they wanted to get pregnant at the time of conception” excluded from the analysis or coded as “no”. Please clarify in this section, as ambivalence about pregnancy intentions may also play a role in women’s receipt of CoC and could potentially be explored separately (if distinct from the women included in this analysis). | The response option was either Yes or No in this question mentioned. We have now clarified this in the explanatory variable section. |
| The authors note, “The factors were selected by using forward regression analysis on the list of factors found in our literature search.” Could they please reference the specific literature from which they determined the initial set of covariates for exploration? Other work on this topic that has found significant associations or conceptual models that indicate the relevance of these factors? Particularly for the partner factors, such as occupation? | We have now added references from which the initial set of covariates for exploration were based. |
| **Results** |  |
| Table 2: I find the presentation of data in Table 2 confusing, particularly because the authors use the terminology “dropout” to describe women’s trajectories through the CoC with ANC as the starting point. As it is currently presented, the authors have provided row totals by delivery and postnatal care status. To improve clarity:  a. Keep the current table format and use column totals, but simplify the row labels. For example, instead of “Delivery care” the section header would be “Delivery by skilled birth attendant”, and the rows beneath it would be “Yes” and “No”. The two rows in that section would sum to 100% for each the ANC <4 and ANC 4+ sub-populations represented by each of the columns. This would provide a clearer narrative to compare women who began the CoC trajectory with 4+ ANC visits, relative to those who did not. b. If the authors do not want to explicitly compare these two populations (by ANC status), they should simplify the table by just including overall proportions of ANC, SBA, and PNC care for the entire sample. c. I also suggest removing the “level of CoC” from each cell in the table, as this is confusing. The second portion of the table that lays out the levels of the continuum is a much clearer presentation. Once this table is revised, the prose explaining the descriptive findings of Table 2 and this part of the analysis should be expanded. | As suggested, we have now edited Table 2. |
| 2. Figure 1: This figure includes a lot of important and helpful information for understanding how women “drop out” from receiving one component of the CoC to another; however, there are a lot of words included in each of the transitional squares and arrows. If possible, simplify the figure, so the proportions of women remaining at each stage are clear and do not get lost among all of the words. a. Check grammar throughout the paragraph of results that outlines this figure. b. The authors conclude the paragraph with, “These trends of dropout were similar across pregnancies that were wanted, mistimed and unwanted at conception.” I would suggest emphasizing that the dropout was more pronounced for pregnancies that were unwanted, as illustrated in the figure. | As suggested, we have checked the grammar and edited the figure for clarity. Also, the sentence mentioned through *point b* has been edited *(See first paragraph at page 13 in the revised manuscript for details)-*  *The trends associated with dropout were more pronounced for pregnancies that were unwanted or mistimed than those that were wanted at conception.* |
| 3. Table 5: Prose needs to be reviewed. For example, the authors state, “A pregnancy that was mistimed at conception was found to be 31% (RR 0.69, 95% CI 0.49-0.97) preventive to the highest level of CoC than a wanted pregnancy.” Should be restated in terms of being a reduced risk of achieving or attaining “high CoC” relative to women with wanted pregnancy. Similarly, in stating, “However, the association between mistimed pregnancy and the moderate level of CoC was no longer statistically significant.” The reference for the multinomial model—i.e. relative to no/low level—should be included. | As suggested, we have now edited the relevant sentences. This now looks like *(See second paragraph at page 13 in the revised manuscript for details)-*  -  *A pregnancy that was mistimed at conception was found to be associated with a 31% (RR 0.69, 95% CI 0.49-0.97) reduction in risk of achieving high CoC than a wanted pregnancy. However, the association between mistimed pregnancy and the moderate level of CoC was no longer statistically significant relative to low/no level.* |
| Discussion |  |
| 1. Overall, this discussion section feels incredibly disjointed and disconnected from the paper. It needs substantial work to orient the findings of the paper within the broader context of this research. a. This is particularly needed for those results related to the household and community level factors that were found to have significant associations with women’s risk of achieving CoC. For example, the findings of this study could be more explicitly woven into or contrasted with the findings of other studies noted in the second paragraph. | As per Reviewer 1’s comments we have addressed the cultural issue and have added information for context around household and community level factors. We have revised discussion section thoroughly to make the linkage clearer. The overall structure of the discussion remains unchanged. |
| 2. There are some incomplete sentences in the discussion that make it a bit confusing to follow. Suggest a thorough re-read with a focus on grammar. | We have now reviewed the Discussion section thoroughly and edited it for grammar and revised incomplete sentences. |
| 3. In acknowledging the limitations of the retrospective pregnancy intention measure, used as the primary explanatory variable in this analysis, the authors state, that “any such bias is likely to be random.” I don’t think I agree with this statement, particularly because the authors do not adjust for time since pregnancy in their analysis.  a. For example, women with index pregnancies occurring earlier than others may be more likely to retrospectively report the pregnancy as intended, now that they have a young child. In contrast, women who have a newborn or infant and are reporting on a much more recent pregnancy may be more likely to report that pregnancy as initially unintended, since the event is more proximate. I think the authors should expand here or in the methods section about how and why this measure is considered valid, despite some of these limitations. | As suggested, we have now edited the explanatory variable section under the methods and added details about how and why unintended pregnancy measure is considered valid. Please see our response on your second comment in the explanatory variable section. |
| **Conclusion** |  |
| 1. In the conclusion, the authors claim, “To ensure CoC, particularly among women who have unintended conceptions, early detection of pregnancy is required, as well as policies to enhance healthcare services at the community level, along with the focus on demand and supply-side interventions together.” However, in the discussion, they emphasize a lack of data about the detection of the pregnancy to be a limitation. How are the authors making a claim about the importance of “early detection of pregnancy”, if this was not central to or even captured in the survey or analysis? | We have now revised this sentence which now has the following read *(See first paragraph at page 21 in the revised manuscript for details)-*  *To ensure CoC, particularly among women who have unintended conceptions, policies are required to enhance healthcare services at the community level, along with the focus on demand- and supply-side interventions together. It is also important for policies to integrate family planning and maternal healthcare services.* |
| 2. The claim that, “Following adjustment of a comprehensive range of individual- , household-, and community-level factors, unintended pregnancy at conception was found to be the strongest preventive predictor of CoC,” seems unsubstantiated by the findings. While pregnancy intention was strongly associated with level of CoC attained, many other factors at the household-level, for example wealth, were found to be the strongest predictors of achieving highest CoC (i.e. RR=3.34 for richest vs. poorest women). Please rephrase the conclusion to reflect the findings more accurately. | We have now edited the relevant sentences which read *(See first paragraph at page 21 in the revised manuscript for details*) -  *Following adjustment for a comprehensive range of individual-, household-, and community-level factors, unintended pregnancy at conception was found to be strongly associated with the level of CoC attained. Different individual-, household-, and community-level factors,* *including lower women’s education, lower household socio-economic status, and lower use of ANC and delivery care services at the community level were also found to be influential determinants of lowering CoC.* |
