# Supplementary material for: Assessing the effect of pregnancy intention at conception on the continuum of care in maternal healthcare services use in Bangladesh: Evidence from a nationally representative cross-sectional survey
Source: PLoS One. 2020 Nov 20;15(11):e0242729. doi: 10.1371/journal.pone.0242729 (PMC7678970; doi:10.1371/journal.pone.0242729)
Supplement: Attachment — Submitted filename: Response to Editor and Reviewer.docx [file pone.0242729.s002.docx]

| **Comments and suggested changes** | **Changes made and authors’ reply** |
| --- | --- |
| Editor’s comment |  |
| Particular attention should be paid to ensuring that the concerns about the proper interpretation and use of literature are addressed.  Secondly, the reviewer suggests removing the following language from the limitations section "However,  any such bias is likely to be random".  The editor strongly agrees.  It is not clear what empirical evidence  suggests this would be the case and theoretically, this assumption seems tenuous as education, age, parity, wealth, and autonomy may all be related to both experiencing an unwanted/unintended pregnancy and how the question is understood and retrospectively  reported. | We have provided the proper interpretation of the terms that the reviewer’s mentioned and updated the citations. We also deleted the sentence referred. |
| **Reviewer#2** |  |
| Overall, the authors have done a good job revising the manuscript according to reviewers’ feedback and have addressed the vast majority of my initial concerns. I have a few final comments/suggestions that I hope will improve the quality of this work.  One remaining concern I have is about the framing and acknowledgement of limitations of the main exposure variable (unintended pregnancy), which both reviewers flagged as an issue in the original submission. In the revised submission, the authors have tried to integrate such limitations into the methods and limitations, but I still have a few concerns with how this is addressed. | We have edited the manuscript in line with your suggestions. The itemized responses are provided below. |
| In the methods, they state, “Importantly, these questions were asked together with contraception use or non-use at that time. Therefore, potential ambivalence associated with a retrospective approach to data collection was reduced greatly as women were able to classify more accurately whether this pregnancy was due to not using contraception because of hoping for a child, contraception failure, or lack of access or use of contraception. The general validity of this measure of data collection and classification at the aggregate level has been repeatedly demonstrated in different settings, including Asian and African countries [33-37].”  • I’m not sure I follow the rationale about how questions about pregnancy intention being asked in the context of contraceptive use would actually improve their validity as measures of pregnancy intendedness. In fact, multiple studies have found that pregnancy intention is inconsistently related to contraceptive use—that is, women may not be using contraception consistently aligned with whether and when they want to become pregnant. • Additionally, the five articles referenced do not accurately address the issue of validity and potential limitations of the pregnancy intention measure. Other articles that specifically examine the validity of different measures of pregnancy intention should be included instead.  o The NIPORT (2016) report and the Hubacher (2008) article referenced do not include any assessment of validity of measures of pregnancy intention (to my knowledge) and should be removed. o The Curtis (2011) article referenced includes a much more nuanced discussion of the ways that pregnancy intendedness relates to contraceptive use, thus, this should be reflected in the current paper if cited. | We have deleted the relevant sentences.  We have deleted the reviewer’s mentioned references and add more new references. |
| Specifically, in the limitations, they state, “An intended pregnancy could also be reported as “unintended” (or “unwanted”) if the outcome of the pregnancy (the child’s sex) is the opposite of the parents’ desire for a child of a particular sex. Moreover, retrospective responses make distinguishing between mistimed and unwanted conception challenging as many women might be unable to determine whether an unintended pregnancy was mistimed or unwanted. However, any such bias is likely to be random.”  • The first sentence: Could the authors expand on or provide literature to justify the mention of sex-specific changes in unintended pregnancy reporting? While valid, it would be helpful to have some literature and/or further explanation for this possible outcome.  • The middle sentence: Could the authors clarify what they mean about how mistimed and unwanted pregnancies may be more difficult due to the retrospective nature of the measure? This feels unclear as currently described. I think the point that they’re trying to make is about the potential nuance of stating a pregnancy is mistimed vs. unwanted many months or years after the pregnancy occurred, simply due to lapsed time, but this is not clearly stated.  • The last sentence: Ending in, “such bias is likely to be random” should be removed, as we do not know this is the case and both reviewers emphasized that this point about measures—especially since this is the main exposure in the analysis—should be framed very clearly as a limitation. | A few changes has made to address these issues that we have mentioned below against each point raised.  We have added two more sentences to justify the sex-specific changes of unintended pregnancy reporting. This reads-  *For instance, an Indian study reported a higher likelihood of unintended pregnancy among women whose child was female than male [72], though, no study in LMICs reported the age-specific changes of unintended pregnancy reporting. This could be because of parents’ higher preference of a male than a female child- a context which is also common in Bangladesh [11].*  We have revised this sentence to address this issue. The revised sentence now reads-  *Moreover, retrospective responses make distinguishing between mistimed and unwanted conception challenging because of the potential nuance of stating a pregnancy is mistimed or unwanted following up to 3 years of conception occurred due to elapsed time.*  *We have deleted this sentence.* |
