# Supplementary material for: Assessing the effect of pregnancy intention at conception on the continuum of care in maternal healthcare services use in Bangladesh: Evidence from a nationally representative cross-sectional survey
Source: PLoS One. 2020 Nov 20;15(11):e0242729. doi: 10.1371/journal.pone.0242729 (PMC7678970; doi:10.1371/journal.pone.0242729)
Supplement: Peer review history

# Peer review: Decision Letter 0

**DOI:** 10.1371/journal.pone.0242729.r001

**Authors:** Linnea A Zimmerman

20 Jul 2020

PONE-D-20-07773

Assessing the effect of pregnancy intention at conception on the continuum of care in maternal healthcare services use in Bangladesh: evidence from a nationally representative cross-sectional survey

PLOS ONE

Dear Dr. Khan,

Thank you for submitting your manuscript to PLOS ONE. After careful consideration, we feel that it has merit but does not fully meet PLOS ONE’s publication criteria as it currently stands. Therefore, we invite you to submit a revised version of the manuscript that addresses the points raised during the review process.

Both reviewers highlight a number of minor suggestions and clarifications.  Please review and respond ​to the suggestions made.  Please ensure that a flow-chart is included per the second reviewer's recommendation. 

Additionally, there are some minor editorial and grammar issues.  PLOS ONE does not support editorial services so your manuscript should be reviewed and edited prior to re-submission to address occasional English language mistakes.

Also ensure that decimal points are consistent throughout the manuscript.  At different points, numbers are presented with zero, one, or two decimal points in the text and tables. Unless using language such as "about 15%", please ensure consistency. 

Please submit your revised manuscript by Sep 03 2020 11:59PM. If you will need more time than this to complete your revisions, please reply to this message or contact the journal office at plosone@plos.org. When you're ready to submit your revision, log on to https://www.editorialmanager.com/pone/ and select the 'Submissions Needing Revision' folder to locate your manuscript file.

Please include the following items when submitting your revised manuscript:

- A rebuttal letter that responds to each point raised by the academic editor and reviewer(s). You should upload this letter as a separate file labeled 'Response to Reviewers'.
- A marked-up copy of your manuscript that highlights changes made to the original version. You should upload this as a separate file labeled 'Revised Manuscript with Track Changes'.
- An unmarked version of your revised paper without tracked changes. You should upload this as a separate file labeled 'Manuscript'.

If you would like to make changes to your financial disclosure, please include your updated statement in your cover letter. Guidelines for resubmitting your figure files are available below the reviewer comments at the end of this letter.

If applicable, we recommend that you deposit your laboratory protocols in protocols.io to enhance the reproducibility of your results. Protocols.io assigns your protocol its own identifier (DOI) so that it can be cited independently in the future. For instructions see: http://journals.plos.org/plosone/s/submission-guidelines#loc-laboratory-protocols

We look forward to receiving your revised manuscript.

Kind regards,

Linnea A Zimmerman, Ph.D, MPH

Academic Editor

PLOS ONE

Journal Requirements:

When submitting your revision, we need you to address these additional requirements.

1. Please ensure that your manuscript meets PLOS ONE's style requirements, including those for file naming. The PLOS ONE style templates can be found at

https://journals.plos.org/plosone/s/file?id=wjVg/PLOSOne_formatting_sample_main_body.pdf and

https://journals.plos.org/plosone/s/file?id=ba62/PLOSOne_formatting_sample_title_authors_affiliations.pdf

2. Thank you for stating the following in the Acknowledgments Section of your manuscript:

'Funding: The first author is supported by a PhD research scholarship from The University of Newcastle, Australia. Dr Melissa L. Harris is supported by an Australian Research Council Discovery Early Career Researcher Award.'

We note that you have provided funding information that is not currently declared in your Funding Statement. However, funding information should not appear in the Acknowledgments section or other areas of your manuscript. We will only publish funding information present in the Funding Statement section of the online submission form.

a. Please remove any funding-related text from the manuscript and let us know how you would like to update your Funding Statement. Currently, your Funding Statement reads as follows:

'The author(s) received no specific funding for this work.'

b. Please include your amended statements within your cover letter; we will change the online submission form on your behalf.

[Note: HTML markup is below. Please do not edit.]

Reviewers' comments:

Reviewer's Responses to Questions

**Comments to the Author**

1. Is the manuscript technically sound, and do the data support the conclusions?

The manuscript must describe a technically sound piece of scientific research with data that supports the conclusions. Experiments must have been conducted rigorously, with appropriate controls, replication, and sample sizes. The conclusions must be drawn appropriately based on the data presented. 

Reviewer #1: Yes

Reviewer #2: Yes

**********

2. Has the statistical analysis been performed appropriately and rigorously? 

Reviewer #1: Yes

Reviewer #2: Yes

**********

3. Have the authors made all data underlying the findings in their manuscript fully available?

The PLOS Data policy requires authors to make all data underlying the findings described in their manuscript fully available without restriction, with rare exception (please refer to the Data Availability Statement in the manuscript PDF file). The data should be provided as part of the manuscript or its supporting information, or deposited to a public repository. For example, in addition to summary statistics, the data points behind means, medians and variance measures should be available. If there are restrictions on publicly sharing data—e.g. participant privacy or use of data from a third party—those must be specified.

Reviewer #1: No

Reviewer #2: Yes

**********

4. Is the manuscript presented in an intelligible fashion and written in standard English?

PLOS ONE does not copyedit accepted manuscripts, so the language in submitted articles must be clear, correct, and unambiguous. Any typographical or grammatical errors should be corrected at revision, so please note any specific errors here.

Reviewer #1: Yes

Reviewer #2: Yes

**********

5. Review Comments to the Author

Please use the space provided to explain your answers to the questions above. You may also include additional comments for the author, including concerns about dual publication, research ethics, or publication ethics. (Please upload your review as an attachment if it exceeds 20,000 characters)

Reviewer #1: This paper investigates the association between having an unintended birth and maternal use of healthcare – specifically continuity of care during and after pregnancy – among women in Bangladesh. The strengths of the paper lie in the large sample, completeness of the measure of unintended births (unwanted and mistimed), and the clear writing. I also want to commend the authors on Figure 1, which provides a very nice depiction of the percentages of women who completed CoC or dropped out. Despite these strengths, some areas of the paper would benefit from additional revision. I include several questions and suggestions below in the aim of strengthening the paper.

One limitation of the current study is that the authors rely solely on retrospective data. This needs to be clarified when describing the data (pg. 8). Additionally, there is a robust literature on the biases inherent in retrospective measures that should be considered in the discussion section (pg. 17) to ascertain what type of bias may have arisen due to this measurement strategy. Many studies from a variety of global settings have found that shifts in women’s reports of intentions are predictable – and tied to intendedness - rather than random as is suggested in the paper. Another potential source of bias is selection into unintended pregnancy. How might this influence the results? Other studies have used analytic strategies like propensity score weighting to control for selection into unintended pregnancy. Although I do not believe that is necessarily required here, the authors should at least acknowledge this source of bias and discuss how it might influence their results.

In the Outcome Variable section, it is important to include the actual survey questions used to create the CoC measure – similar to what is done in the Explanatory variables section (see pg. 8). This would clear up several of my questions about the items used to construct this measure, such as how a skilled birth attendant is defined.

In the literature review, the unintended fertility rate in Bangladesh is described as being 46% of all pregnancies (pg. 6), but the current study finds that only 26% of births are unintended. Please include a discussion of why you believe there is such a large difference between these percentages (selection? Measuring pregnancies vs. births?), and describe how this may have affected your results and therefore your conclusions.

The authors claim that they are able to make the “strongest recommendations for future policies” (pg. 18), but from my reading, many of the policy recommendations are rather vague. This is the area of the discussion that needs the most attention. For example, one of the authors’ recommendations is to create policies that “integrate healthcare services” (pg. 14). Can the authors provide some concrete suggestions on how Bangladesh – or LMICs more broadly – might do this and/or what these policies might look like? Similar questions apply to the recommendation for policies to “consider a range of factors…” (pg. 15). Addressing these questions and providing concrete policy recommendations would greatly strengthen the paper and its potential to contribute to positive changes in maternal health services and utilization in Bangladesh.

One of the study’s findings is that Bangladesh falls in the middle in terms of its dropout rate. I’d like to see the authors offer some explanations of why they think this is the case. Also, what could Bangladesh do better to emulate the South Asian countries mentioned on page 14?

Cultural factors are mentioned as a barrier to increasing CoC among women in Bangladesh. How might these be addressed in policy creation and implementation? Addressing this would strengthen the discussion on page 16.

Reviewer #2: This study investigates an important issue – continuum of pregnancy-related care (CoC) in low and lower-middle income countries and the impact that unintended pregnancy has on receipt of care. This work is particularly important for recognizing the potential disparities and opportunities for intervention in improving receipt of recommended maternal health services, particularly among women who experience unintended pregnancy. Below are additional comments that hopefully will improve the contribution that this article can make, mostly in terms of the presentation and framing of the dependent variable and reporting results. Overall, this is a thoughtful article, which extends the current body of maternity care research beyond simple measures of ANC, SBA and PNC.

Abstract

1. Methods: It would be helpful if the authors could note the data source (DHS Bangladesh 2014) in the abstract, so readers understand the type of dataset used for the analysis.

2. Results: The language about “maintaining” vs. “achieving” CoC is confusing. Per the definition of the CoC (high, moderate, and low/none), the measure is complete when all components of pregnancy-related care are considered. Use of the word “maintain” here makes it seem as though the study examines CoC as a measure over time, instead of within the context of a single index pregnancy. Suggest to reword any use of “maintain” as “achieve/attain” throughout the abstract and manuscript for clarity.

Methods

1. Data:

a. The subsample of women included in this analysis must have had a livebirth in three years prior to the survey; responded to the question on maternity care, and reported retrospective pregnancy intention. What proportion of women aged 15-49 had a birth in the prior three years, but did not have complete measures on all three components of CoC? It might be interesting, or at least provide some insight, if the authors could note which components of those eligibility criteria resulted in exclusion (i.e. some kind of flowchart with numbers of how they reached the final sample, for reference). Unclear if the jump from 17,863 to 4,493 women was due to a relatively low number of women who had a birth within the three years prior; incomplete data on maternal health services; or no pregnancy intention reported at conception, which are relevant for understanding the broader study sample from which this analytic sample was derived.

2. Outcome variable:

a. The authors indicate that they followed the WHO’s 2014 guidelines for maternity care, including receipt of at least four ANC visits. They also note that this changed to eight in 2016. Why did the authors choose to assess 4+ visits instead of 8+ visits, given the change in recommendations? Were any sensitivity analyses conducted to see how this altered the distribution of outcomes? I suspect far fewer women would have achieved a “high” level of continuum of care, but might provide an interesting comparison at least to address in the discussion.

b. While the authors note that their development of the outcome variable aligns with the WHO’s guidance, I would like to know more about their decision to “weight” all three components of care equally. For example, is a woman who received ANC and PNC, but no SBA likely to encounter different health risks than women who received a different combination of care (e.g., ANC, SBA, no PNC)? Do the authors anticipate that this would have altered the outcome?

c. Throughout the paper, the authors use the terminology “dropout” to describe women who received early components of CoC, but did not receive later components. This language, which reframes the dependent variable to consider each “stage” of the CoC, should be described in this part of the methods section. As a reviewer, it was not clear in the methods that the analysis would include more nuanced assessments of the CoC, other than the three-level categorical variable that is described.

3. Explanatory variables

a. It would be helpful if the authors clearly stated that “women’s intention at conception” was the woman’s fertility/pregnancy intention. The term “intention at conception” is a bit abstract; “index pregnancy intention” may be a better term for this concept throughout the manuscript.

b. Authors should also indicate that this is a retrospectively reported measure, one that may be reporting on a pregnancy from up to three years prior. Limitations of such a measure should be noted.

c. Were women who responded “Don’t know” to the question of “whether they wanted to get pregnant at the time of conception” excluded from the analysis or coded as “no”. Please clarify in this section, as ambivalence about pregnancy intentions may also play a role in women’s receipt of CoC and could potentially be explored separately (if distinct from the women included in this analysis).

d. The authors note, “The factors were selected by using forward regression analysis on the list of factors found in our literature search.” Could they please reference the specific literature from which they determined the initial set of covariates for exploration? Other work on this topic that has found significant associations or conceptual models that indicate the relevance of these factors? Particularly for the partner factors, such as occupation?

Results

1. Table 2: I find the presentation of data in Table 2 confusing, particularly because the authors use the terminology “dropout” to describe women’s trajectories through the CoC with ANC as the starting point. As it is currently presented, the authors have provided row totals by delivery and postnatal care status. To improve clarity:

a. Keep the current table format and use column totals, but simplify the row labels. For example, instead of “Delivery care” the section header would be “Delivery by skilled birth attendant”, and the rows beneath it would be “Yes” and “No”. The two rows in that section would sum to 100% for each the ANC <4 and ANC 4+ sub-populations represented by each of the columns. This would provide a clearer narrative to compare women who began the CoC trajectory with 4+ ANC visits, relative to those who did not.

b. If the authors do not want to explicitly compare these two populations (by ANC status), they should simplify the table by just including overall proportions of ANC, SBA, and PNC care for the entire sample.

c. I also suggest removing the “level of CoC” from each cell in the table, as this is confusing. The second portion of the table that lays out the levels of the continuum is a much clearer presentation. Once this table is revised, the prose explaining the descriptive findings of Table 2 and this part of the analysis should be expanded.

2. Figure 1: This figure includes a lot of important and helpful information for understanding how women “drop out” from receiving one component of the CoC to another; however, there are a lot of words included in each of the transitional squares and arrows. If possible, simplify the figure, so the proportions of women remaining at each stage are clear and do not get lost among all of the words.

a. Check grammar throughout the paragraph of results that outlines this figure.

b. The authors conclude the paragraph with, “These trends of dropout were similar across pregnancies that were wanted, mistimed and unwanted at conception.” I would suggest emphasizing that the dropout was more pronounced for pregnancies that were unwanted, as illustrated in the figure.

3. Table 5: Prose needs to be reviewed. For example, the authors state, “A pregnancy that was mistimed at conception was found to be 31% (RR 0.69, 95% CI 0.49-0.97) preventive to the highest level of CoC than a wanted pregnancy.” Should be restated in terms of being a reduced risk of achieving or attaining “high CoC” relative to women with wanted pregnancy. Similarly, in stating, “However, the association between mistimed pregnancy and the moderate level of CoC was no longer statistically significant.” The reference for the multinomial model—i.e. relative to no/low level—should be included.

Discussion

1. Overall, this discussion section feels incredibly disjointed and disconnected from the paper. It needs substantial work to orient the findings of the paper within the broader context of this research.

a. This is particularly needed for those results related to the household and community level factors that were found to have significant associations with women’s risk of achieving CoC. For example, the findings of this study could be more explicitly woven into or contrasted with the findings of other studies noted in the second paragraph.

2. There are some incomplete sentences in the discussion that make it a bit confusing to follow. Suggest a thorough re-read with a focus on grammar.

3. In acknowledging the limitations of the retrospective pregnancy intention measure, used as the primary explanatory variable in this analysis, the authors state, that “any such bias is likely to be random.” I don’t think I agree with this statement, particularly because the authors do not adjust for time since pregnancy in their analysis.

a. For example, women with index pregnancies occurring earlier than others may be more likely to retrospectively report the pregnancy as intended, now that they have a young child. In contrast, women who have a newborn or infant and are reporting on a much more recent pregnancy may be more likely to report that pregnancy as initially unintended, since the event is more proximate. I think the authors should expand here or in the methods section about how and why this measure is considered valid, despite some of these limitations.

Conclusion

1. In the conclusion, the authors claim, “To ensure CoC, particularly among women who have unintended conceptions, early detection of pregnancy is required, as well as policies to enhance healthcare services at the community level, along with the focus on demand and supply-side interventions together.” However, in the discussion, they emphasize a lack of data about the detection of the pregnancy to be a limitation. How are the authors making a claim about the importance of “early detection of pregnancy”, if this was not central to or even captured in the survey or analysis?

2. The claim that, “Following adjustment of a comprehensive range of individual- , household-, and community-level factors, unintended pregnancy at conception was found to be the strongest preventive predictor of CoC,” seems unsubstantiated by the findings. While pregnancy intention was strongly associated with level of CoC attained, many other factors at the household-level, for example wealth, were found to be the strongest predictors of achieving highest CoC (i.e. RR=3.34 for richest vs. poorest women). Please rephrase the conclusion to reflect the findings more accurately.

**********

6. PLOS authors have the option to publish the peer review history of their article (what does this mean?). If published, this will include your full peer review and any attached files.

If you choose “no”, your identity will remain anonymous but your review may still be made public.

**Do you want your identity to be public for this peer review?** For information about this choice, including consent withdrawal, please see our Privacy Policy.

Reviewer #1: No

Reviewer #2: No

[NOTE: If reviewer comments were submitted as an attachment file, they will be attached to this email and accessible via the submission site. Please log into your account, locate the manuscript record, and check for the action link "View Attachments". If this link does not appear, there are no attachment files.]

While revising your submission, please upload your figure files to the Preflight Analysis and Conversion Engine (PACE) digital diagnostic tool, https://pacev2.apexcovantage.com/. PACE helps ensure that figures meet PLOS requirements. To use PACE, you must first register as a user. Registration is free. Then, login and navigate to the UPLOAD tab, where you will find detailed instructions on how to use the tool. If you encounter any issues or have any questions when using PACE, please email PLOS at figures@plos.org. Please note that Supporting Information files do not need this step.

# Author comment: Author response to Decision Letter 0

**DOI:** 10.1371/journal.pone.0242729.r002

18 Aug 2020

Please see the relevant attached file- one file added there to respond the reviewers' comments.

Attachment Submitted filename: Response to Reviewers.docx Click here for additional data file.

# Peer review: Decision Letter 1

**DOI:** 10.1371/journal.pone.0242729.r003

**Authors:** Linnea A Zimmerman

9 Oct 2020

PONE-D-20-07773R1

Assessing the effect of pregnancy intention at conception on the continuum of care in maternal healthcare services use in Bangladesh: evidence from a nationally representative cross-sectional survey

PLOS ONE

Dear Dr. Khan,

Thank you for submitting your manuscript to PLOS ONE. After careful consideration, we feel that it has merit but does not fully meet PLOS ONE’s publication criteria as it currently stands. Therefore, we invite you to submit a revised version of the manuscript that addresses the points raised during the review process.

While one of the reviewer's found the previous edits acceptable, the second felt that there were minor revisions that were still required, with which the editor agrees.  Please review the comments attached.

Particular attention should be paid to ensuring that the concerns about the proper interpretation and use of literature are addressed.  Secondly, the reviewer suggests removing the following language from the limitations section "However,  any such bias is likely to be random".  The editor strongly agrees.  It is not clear what empirical evidence  suggests this would be the case and theoretically, this assumption seems tenuous as education, age, parity, wealth, and autonomy may all be related to both experiencing an unwanted/unintended pregnancy and how the question is understood and retrospectively  reported.

Please submit your revised manuscript by Nov 23 2020 11:59PM. If you will need more time than this to complete your revisions, please reply to this message or contact the journal office at plosone@plos.org. When you're ready to submit your revision, log on to https://www.editorialmanager.com/pone/ and select the 'Submissions Needing Revision' folder to locate your manuscript file.

Please include the following items when submitting your revised manuscript:

- A rebuttal letter that responds to each point raised by the academic editor and reviewer(s). You should upload this letter as a separate file labeled 'Response to Reviewers'.
- A marked-up copy of your manuscript that highlights changes made to the original version. You should upload this as a separate file labeled 'Revised Manuscript with Track Changes'.
- An unmarked version of your revised paper without tracked changes. You should upload this as a separate file labeled 'Manuscript'.

If you would like to make changes to your financial disclosure, please include your updated statement in your cover letter. Guidelines for resubmitting your figure files are available below the reviewer comments at the end of this letter.

If applicable, we recommend that you deposit your laboratory protocols in protocols.io to enhance the reproducibility of your results. Protocols.io assigns your protocol its own identifier (DOI) so that it can be cited independently in the future. For instructions see: http://journals.plos.org/plosone/s/submission-guidelines#loc-laboratory-protocols

We look forward to receiving your revised manuscript.

Kind regards,

Linnea A Zimmerman, Ph.D, MPH

Academic Editor

PLOS ONE

[Note: HTML markup is below. Please do not edit.]

Reviewers' comments:

Reviewer's Responses to Questions

**Comments to the Author**

1. If the authors have adequately addressed your comments raised in a previous round of review and you feel that this manuscript is now acceptable for publication, you may indicate that here to bypass the “Comments to the Author” section, enter your conflict of interest statement in the “Confidential to Editor” section, and submit your "Accept" recommendation.

Reviewer #1: All comments have been addressed

Reviewer #2: (No Response)

**********

2. Is the manuscript technically sound, and do the data support the conclusions?

The manuscript must describe a technically sound piece of scientific research with data that supports the conclusions. Experiments must have been conducted rigorously, with appropriate controls, replication, and sample sizes. The conclusions must be drawn appropriately based on the data presented. 

Reviewer #1: Yes

Reviewer #2: Yes

**********

3. Has the statistical analysis been performed appropriately and rigorously? 

Reviewer #1: Yes

Reviewer #2: Yes

**********

4. Have the authors made all data underlying the findings in their manuscript fully available?

The PLOS Data policy requires authors to make all data underlying the findings described in their manuscript fully available without restriction, with rare exception (please refer to the Data Availability Statement in the manuscript PDF file). The data should be provided as part of the manuscript or its supporting information, or deposited to a public repository. For example, in addition to summary statistics, the data points behind means, medians and variance measures should be available. If there are restrictions on publicly sharing data—e.g. participant privacy or use of data from a third party—those must be specified.

Reviewer #1: Yes

Reviewer #2: Yes

**********

5. Is the manuscript presented in an intelligible fashion and written in standard English?

PLOS ONE does not copyedit accepted manuscripts, so the language in submitted articles must be clear, correct, and unambiguous. Any typographical or grammatical errors should be corrected at revision, so please note any specific errors here.

Reviewer #1: Yes

Reviewer #2: Yes

**********

6. Review Comments to the Author

Please use the space provided to explain your answers to the questions above. You may also include additional comments for the author, including concerns about dual publication, research ethics, or publication ethics. (Please upload your review as an attachment if it exceeds 20,000 characters)

Reviewer #1: (No Response)

Reviewer #2: Overall, the authors have done a good job revising the manuscript according to reviewers’ feedback and have addressed the vast majority of my initial concerns. I have a few final comments/suggestions that I hope will improve the quality of this work.

One remaining concern I have is about the framing and acknowledgement of limitations of the main exposure variable (unintended pregnancy), which both reviewers flagged as an issue in the original submission. In the revised submission, the authors have tried to integrate such limitations into the methods and limitations, but I still have a few concerns with how this is addressed.

In the methods, they state, “Importantly, these questions were asked together with contraception use or non-use at that time. Therefore, potential ambivalence associated with a retrospective approach to data collection was reduced greatly as women were able to classify more accurately whether this pregnancy was due to not using contraception because of hoping for a child, contraception failure, or lack of access or use of contraception. The general validity of this measure of data collection and classification at the aggregate level has been repeatedly demonstrated in different settings, including Asian and African countries [33-37].”

• I’m not sure I follow the rationale about how questions about pregnancy intention being asked in the context of contraceptive use would actually improve their validity as measures of pregnancy intendedness. In fact, multiple studies have found that pregnancy intention is inconsistently related to contraceptive use—that is, women may not be using contraception consistently aligned with whether and when they want to become pregnant.

• Additionally, the five articles referenced do not accurately address the issue of validity and potential limitations of the pregnancy intention measure. Other articles that specifically examine the validity of different measures of pregnancy intention should be included instead.

o The NIPORT (2016) report and the Hubacher (2008) article referenced do not include any assessment of validity of measures of pregnancy intention (to my knowledge) and should be removed.

o The Curtis (2011) article referenced includes a much more nuanced discussion of the ways that pregnancy intendedness relates to contraceptive use, thus, this should be reflected in the current paper if cited.

Specifically, in the limitations, they state, “An intended pregnancy could also be reported as “unintended” (or “unwanted”) if the outcome of the pregnancy (the child’s sex) is the opposite of the parents’ desire for a child of a particular sex. Moreover, retrospective responses make distinguishing between mistimed and unwanted conception challenging as many women might be unable to determine whether an unintended pregnancy was mistimed or unwanted. However, any such bias is likely to be random.”

• The first sentence: Could the authors expand on or provide literature to justify the mention of sex-specific changes in unintended pregnancy reporting? While valid, it would be helpful to have some literature and/or further explanation for this possible outcome.

• The middle sentence: Could the authors clarify what they mean about how mistimed and unwanted pregnancies may be more difficult due to the retrospective nature of the measure? This feels unclear as currently described. I think the point that they’re trying to make is about the potential nuance of stating a pregnancy is mistimed vs. unwanted many months or years after the pregnancy occurred, simply due to lapsed time, but this is not clearly stated.

• The last sentence: Ending in, “such bias is likely to be random” should be removed, as we do not know this is the case and both reviewers emphasized that this point about measures—especially since this is the main exposure in the analysis—should be framed very clearly as a limitation.

**********

7. PLOS authors have the option to publish the peer review history of their article (what does this mean?). If published, this will include your full peer review and any attached files.

If you choose “no”, your identity will remain anonymous but your review may still be made public.

**Do you want your identity to be public for this peer review?** For information about this choice, including consent withdrawal, please see our Privacy Policy.

Reviewer #1: No

Reviewer #2: No

[NOTE: If reviewer comments were submitted as an attachment file, they will be attached to this email and accessible via the submission site. Please log into your account, locate the manuscript record, and check for the action link "View Attachments". If this link does not appear, there are no attachment files.]

While revising your submission, please upload your figure files to the Preflight Analysis and Conversion Engine (PACE) digital diagnostic tool, https://pacev2.apexcovantage.com/. PACE helps ensure that figures meet PLOS requirements. To use PACE, you must first register as a user. Registration is free. Then, login and navigate to the UPLOAD tab, where you will find detailed instructions on how to use the tool. If you encounter any issues or have any questions when using PACE, please email PLOS at figures@plos.org. Please note that Supporting Information files do not need this step.

# Author comment: Author response to Decision Letter 1

**DOI:** 10.1371/journal.pone.0242729.r004

10 Oct 2020

A relevant file is attached where we have provided item by item response to the editor and reviewer's comments.

Attachment Submitted filename: Response to Editor and Reviewer.docx Click here for additional data file.

# Peer review: Decision Letter 2

**DOI:** 10.1371/journal.pone.0242729.r005

**Authors:** Linnea A Zimmerman

9 Nov 2020

Assessing the effect of pregnancy intention at conception on the continuum of care in maternal healthcare services use in Bangladesh: evidence from a nationally representative cross-sectional survey

PONE-D-20-07773R2

Dear Dr. Khan,

We’re pleased to inform you that your manuscript has been judged scientifically suitable for publication and will be formally accepted for publication once it meets all outstanding technical requirements.

Within one week, you’ll receive an e-mail detailing the required amendments. When these have been addressed, you’ll receive a formal acceptance letter and your manuscript will be scheduled for publication.

An invoice for payment will follow shortly after the formal acceptance. To ensure an efficient process, please log into Editorial Manager at http://www.editorialmanager.com/pone/, click the 'Update My Information' link at the top of the page, and double check that your user information is up-to-date. If you have any billing related questions, please contact our Author Billing department directly at authorbilling@plos.org.

If your institution or institutions have a press office, please notify them about your upcoming paper to help maximize its impact. If they’ll be preparing press materials, please inform our press team as soon as possible -- no later than 48 hours after receiving the formal acceptance. Your manuscript will remain under strict press embargo until 2 pm Eastern Time on the date of publication. For more information, please contact onepress@plos.org.

Kind regards,

Linnea A Zimmerman, Ph.D, MPH

Academic Editor

PLOS ONE

Additional Editor Comments (optional):

Reviewers' comments:

Reviewer's Responses to Questions

**Comments to the Author**

1. If the authors have adequately addressed your comments raised in a previous round of review and you feel that this manuscript is now acceptable for publication, you may indicate that here to bypass the “Comments to the Author” section, enter your conflict of interest statement in the “Confidential to Editor” section, and submit your "Accept" recommendation.

Reviewer #2: All comments have been addressed

**********

2. Is the manuscript technically sound, and do the data support the conclusions?

The manuscript must describe a technically sound piece of scientific research with data that supports the conclusions. Experiments must have been conducted rigorously, with appropriate controls, replication, and sample sizes. The conclusions must be drawn appropriately based on the data presented. 

Reviewer #2: Yes

**********

3. Has the statistical analysis been performed appropriately and rigorously? 

Reviewer #2: Yes

**********

4. Have the authors made all data underlying the findings in their manuscript fully available?

The PLOS Data policy requires authors to make all data underlying the findings described in their manuscript fully available without restriction, with rare exception (please refer to the Data Availability Statement in the manuscript PDF file). The data should be provided as part of the manuscript or its supporting information, or deposited to a public repository. For example, in addition to summary statistics, the data points behind means, medians and variance measures should be available. If there are restrictions on publicly sharing data—e.g. participant privacy or use of data from a third party—those must be specified.

Reviewer #2: Yes

**********

5. Is the manuscript presented in an intelligible fashion and written in standard English?

PLOS ONE does not copyedit accepted manuscripts, so the language in submitted articles must be clear, correct, and unambiguous. Any typographical or grammatical errors should be corrected at revision, so please note any specific errors here.

Reviewer #2: Yes

**********

6. Review Comments to the Author

Please use the space provided to explain your answers to the questions above. You may also include additional comments for the author, including concerns about dual publication, research ethics, or publication ethics. (Please upload your review as an attachment if it exceeds 20,000 characters)

Reviewer #2: No remaining concerns to address. Authors have made appropriate changes to improve the manuscript through their revisions.

**********

7. PLOS authors have the option to publish the peer review history of their article (what does this mean?). If published, this will include your full peer review and any attached files.

If you choose “no”, your identity will remain anonymous but your review may still be made public.

**Do you want your identity to be public for this peer review?** For information about this choice, including consent withdrawal, please see our Privacy Policy.

Reviewer #2: No

# Editor report: Acceptance letter

**DOI:** 10.1371/journal.pone.0242729.r006

**Authors:** Linnea A Zimmerman

11 Nov 2020

PONE-D-20-07773R2 

Assessing the effect of pregnancy intention at conception on the continuum of care in maternal healthcare services use in Bangladesh: evidence from a nationally representative cross-sectional survey 

Dear Dr. Khan:

I'm pleased to inform you that your manuscript has been deemed suitable for publication in PLOS ONE. Congratulations! Your manuscript is now with our production department. 

If your institution or institutions have a press office, please let them know about your upcoming paper now to help maximize its impact. If they'll be preparing press materials, please inform our press team within the next 48 hours. Your manuscript will remain under strict press embargo until 2 pm Eastern Time on the date of publication. For more information please contact onepress@plos.org.

If we can help with anything else, please email us at plosone@plos.org. 

Thank you for submitting your work to PLOS ONE and supporting open access. 

Kind regards, 

PLOS ONE Editorial Office Staff

on behalf of

Dr. Linnea A Zimmerman 

Academic Editor

PLOS ONE
